# Machine Learning for Risk Group Identification and User Data Collection in a Herpes Simplex Virus Patient Registry: Algorithm Development and Validation Study

**DOI:** 10.2196/25560

**Published:** 2021-06-11

**Authors:** Svitlana Surodina, Ching Lam, Svetislav Grbich, Madison Milne-Ives, Michelle van Velthoven, Edward Meinert

**Affiliations:** 1 Skein Ltd London United Kingdom; 2 Department of Informatics King’s College London London United Kingdom; 3 Institute of Biomedical Engineering Department of Engineering Science University of Oxford Oxford United Kingdom; 4 Centre for Health Technology University of Plymouth Plymouth United Kingdom; 5 Nuffield Department of Primary Health Sciences Medical Sciences Division University of Oxford Oxford United Kingdom; 6 Department of Primary Care and Public Health School of Public Health Imperial College London London United Kingdom

**Keywords:** data collection, herpes simplex virus, registries, machine learning, risk assessment, artificial intelligence, medical information system, user-centered design, predictor, risk

## Abstract

**Background:**

Researching people with herpes simplex virus (HSV) is challenging because of poor data quality, low user engagement, and concerns around stigma and anonymity.

**Objective:**

This project aimed to improve data collection for a real-world HSV registry by identifying predictors of HSV infection and selecting a limited number of relevant questions to ask new registry users to determine their level of HSV infection risk.

**Methods:**

The US National Health and Nutrition Examination Survey (NHANES, 2015-2016) database includes the confirmed HSV type 1 and type 2 (HSV-1 and HSV-2, respectively) status of American participants (14-49 years) and a wealth of demographic and health-related data. The questionnaires and data sets from this survey were used to form two data sets: one for HSV-1 and one for HSV-2. These data sets were used to train and test a model that used a random forest algorithm (devised using Python) to minimize the number of anonymous lifestyle-based questions needed to identify risk groups for HSV.

**Results:**

The model selected a reduced number of questions from the NHANES questionnaire that predicted HSV infection risk with high accuracy scores of 0.91 and 0.96 and high recall scores of 0.88 and 0.98 for the HSV-1 and HSV-2 data sets, respectively. The number of questions was reduced from 150 to an average of 40, depending on age and gender. The model, therefore, provided high predictability of risk of infection with minimal required input.

**Conclusions:**

This machine learning algorithm can be used in a real-world evidence registry to collect relevant lifestyle data and identify individuals’ levels of risk of HSV infection. A limitation is the absence of real user data and integration with electronic medical records, which would enable model learning and improvement. Future work will explore model adjustments, anonymization options, explicit permissions, and a standardized data schema that meet the General Data Protection Regulation, Health Insurance Portability and Accountability Act, and third-party interface connectivity requirements.

## Introduction

### Background

Patient data in medical registries are an important source of information for screening, treatment, and research purposes. However, the value of these registries can be severely limited by a lack of high-quality data, especially in diseases where there are low patient engagement and concerns around stigma and anonymity, such as herpes simplex virus (HSV) [1]. Improving the quality and quantity of data collection from patients with HSV and individuals who may be at risk of HSV would provide significant benefits for research and clinical care. There is an urgent need for a vaccine for HSV type 2 (HSV-2), and its research relies on a collection of relevant data [2]. Large data sets of high-quality data will enable researchers to gain new insights into HSV and clinicians to personalize care plans to improve individuals’ health outcomes and quality of life. HSV registry database design poses unique data quality challenges related to the stigma of the HSV condition, increased privacy concern, and selection biases [3]. For example, the lack of data on people who are living with HSV without symptoms calls to a specific need to collect data outside of clinical settings from populations who have not developed symptoms and are not motivated to complete extensive questionnaires or, worse, take offence at being asked to do so. Therefore, nonintrusive and time-efficient methods are necessary to reliably identify high-risk groups.

New technologies, such as machine learning and artificial intelligence, are gaining traction in medical research as a means of collecting and analyzing data [4,5]. Machine learning has previously been applied to medical diagnosis and patient data insights in oncology [6], for the diagnosis of heart, liver, and diabetic diseases, as well as infectious diseases such as dengue, and hepatitis [7], to predict suicidal behavior using longitudinal electronic health record (EHR) data [8], and to classify whether patients have Alzheimer disease [9]. However, current applications of machine learning are usually limited to pre-existing, structured data sets and do not address the problems of first-person data collection from patients and the resulting limitations of data completeness, quality, and validity.

Machine learning–based systems are particularly useful for generating new knowledge and insights without having a priori hypotheses. The concept of “data farming” or “evidence farming” addresses the problems of collecting data directly from users [10]. Data can be “organic” —grown and harvested—if suitable environmental conditions are provided. These conditions are often met by websites and online platforms where patients provide their data, such as the online enterprise PatientsLikeMe.com [11]. Data collected from these platforms can include medical information like disease diagnosis and laboratory results, medications the patients are taking, and subjective data from self-report questionnaires.

Data generation platforms like these provide an opportunity to gather the large amounts of data needed to develop machine learning models. One type of decision support model, decision trees, can be applied to analyze the flows of user-generated content and to determine the strategy that is most efficient and most likely to successfully achieve a certain goal [12]. Decision tree analyses are widely used in health care, but primarily in a basic way, on highly structured data, without applications in real-time data collection situations [13]. A key limitation of data tree analyses is that they are very sensitive to the data they are trained on [14]. However, machine learning methods such as random forest, which are based on a collection of individual decision trees, can minimize the effect of this limitation [14].

Machine learning methods have great potential in the field of real-world data [15]. They have particular promise for analyzing large “data lakes” that have been created by aggregating information from hospital EHRs, including unstructured and semistructured patient-generated data. Machine learning methods can explore this data to identify clinically meaningful patterns. Because these data lakes can track patients longitudinally, they provide a large body of data that would not be available in the typical randomized controlled trial. Therefore, the application of machine learning to these data sets could reduce, or even eliminate, the need for certain traditionally conducted late-phase trials. Drugs that have successfully completed phase I and II trials, and have evidence supporting their efficacy and safety, could be given to patients and monitored in the context of patients’ real-world experiences [16]. The application of machine learning methods to such real-world databases also provides an opportunity to easily identify potentially eligible patients for clinical trials, by filtering the database based on relevant criteria. The potential value of this real-world data highlights the importance of developing ways of collecting high-quality data from patient registry platforms.

### Challenges of Developing Patient Registries

Patient registries collect longitudinal data about a specific population of interest to provide real-world evidence and insights into disease progression, factors affecting health outcomes and quality of life, and the effectiveness of different treatments [17,18]. Previously developed patient-focused digital registries, such as ArthritisPower, have achieved significant results in terms of patient engagement [19]. The ArthritisPower registry platform has proven to be an effective means of engaging patients to participate in research and enabling patient-generated data capture; however, the registry is limited to users who have already received a physician diagnosis and are actively motivated to participate. In addition to increased patient engagement with research, the growing focus on patient-centered care led to new emphasis on the use of patient-reported outcome measures in clinical care and research, and patient-reported outcomes now constitute a key component of patient registries [20]. Validated questionnaires provide a useful means of collecting standardized data, but the inclusion of multiple questionnaires in a patient registry can result in a long and arduous process for patients [21]. However, self-reported registries have the benefit of privacy and anonymization, which is particularly important for health conditions that people are uncomfortable discussing with clinicians or researchers, and they have significant potential for developing large databases of evidence that can inform clinical care and provide new insights into the condition in question [22].

However, several challenges need to be addressed to develop a usable and effective patient registry [3]:

1. Efficient use of data: collecting sufficient and high-quality data is necessary to provide useful insights and allow for more targeted clinical trial selection and recruitment. However, this tends to require long patient questionnaires, which often results in high drop-out rates. Therefore, a critical challenge for digital patient data collection is to maximize the usefulness, quality, and information content of the collected data while reducing time and effort for users.
2. Patient-centric design: to be usable and effective, patient registry data collection processes need to serve the expectations and needs of the patients. Therefore, another key challenge is to ensure that patients, and their experience, are considered from the early stages of patient registry development.
3. Selection bias: direct data collection methods can increase the risk of selection bias in the data because subsets of users with certain characteristics are more likely to complete an extensive questionnaire (eg, more computer-literate users), have more time, have more frequent or severe symptoms, and/or are keen to be informed of relevant clinical trials. The challenge for patient registries is to minimize the risk of selection bias by facilitating and simplifying the data collection process.
4. Privacy concerns: common user concerns when it comes to digital data collection are related to privacy and control over data. This is especially true for sensitive or personally identifiable information. This is a key challenge that must be addressed to ensure the adoption and sustainability of any digital solution.

### Aim and Objectives

The resolution of the first of the four challenges described above relies on the ability to reduce the drop-off rates by shortening the time and effort required to complete the questionnaire without critically affecting the quality and quantity of results. Similarly, the patient-centric design (challenge 2) requires consideration of user experience, which includes minimizing participant burden. This may also ultimately reduce selection bias (challenge 3) by increasing completion rates. Therefore, this project is aimed primarily at addressing the challenges associated with time-consuming questionnaires containing sensitive questions by creating a prediction model to reliably assess whether a particular person has an increased risk of HSV. We explored the applications of innovative machine learning methods to optimize the questions asked of participants while maintaining the high quality and relevance of collected data. The main objective was to use a random forest model to design an HSV patient registry that can use various lifestyle predictors for HSV infection (eg, sexual activity, number of partners) and recurrences (eg, diet, exercise, sleep) to select the most relevant questions for an HSV registry and improve data collection and analysis in medical registries. For future studies, we suggest integrating this approach with privacy-preserving and trust-enabling solutions to more comprehensively address the four challenges described above. 

### Review of Past Studies

Multiple studies in recent years have sought to create machine learning models based on data from EHRs and other sources applying the multifactor classification approach to assess and predict risk groups for medical conditions and complications and to identify major risk factors. Such targets include delirium occurrences [23], alcohol use disorder [24], mortality in patients with liver disease [25], cardio-cerebrovascular events [26], suicide attempts [27], metabolic syndrome [28], and postpartum depression [29]. Random forest has been discussed as one of the most efficient methods for creating risk prediction models [14,30,31] and has been applied in most of the studies reviewed.

However, the considerations of user-focused design in the context of optimizing direct data collection for an HSV medical registry introduce additional context to the classification problem, including the emotional sensitivity of some questions and the need to minimize the number of factors (questions) in the ensemble to increase completion rates and address the other limitations discussed above. This study aims to apply the random forest classification approach to enable more efficient data collection from the population while providing an effective tool for HSV screening.

### Overview of the Proposed Solution

We designed an algorithm to optimize data collection questionnaires for an HSV patient registry and predict HSV infection risk. Integrating a decision tree–based technology into a patient registry can reduce the number of questions users have to answer while increasing the data content and reducing informational entropy for each user’s record. The model was trained using a pre-existing data set so that the question sets could be optimized to collect initial user data efficiently and generate the most complete information to screen users’ HSV risk. For nondiagnosed users, the model identifies risk groups based on the data provided. Data from users who are clinically diagnosed with HSV can be used to further train and improve the model.

**Figure 1:**
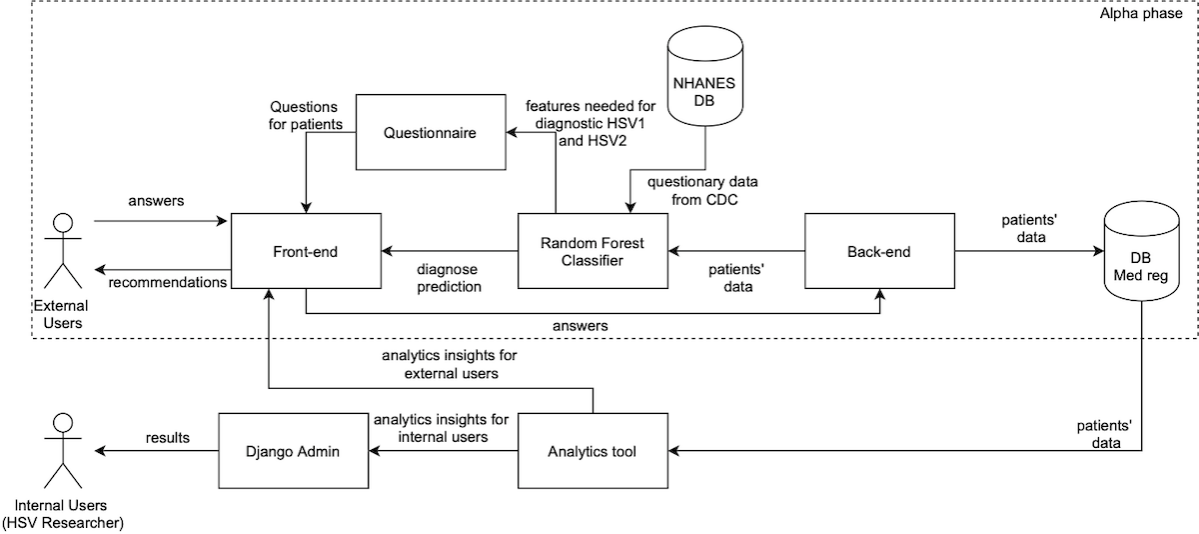
Schema of the technological solution. HSV: herpes simplex virus, NHANES: US National Health and Nutrition Examination Survey, DB: database, CDC: Centers for Disease Control and Prevention, Med reg: medical registry.

HSV screening was chosen as the goal for the prototype solution to provide an efficient way of engaging users who otherwise might not be willing to participate in research or log HSV recurrence data. Three categories of users were considered when designing the system: two types of patient users (new and returning; external users) and HSV researchers (internal users). Since the barriers to user engagement and data collection are most pronounced when an external user starts using the system for the first time, an HSV preliminary screening tool was included to serve as an incentive to answer the list of questions and provide users with an assessment of their risk of HSV. The anonymous questionnaire was optimized to reduce information entropy and minimize the number of questions and their complexity while obtaining the maximum amount of information. The solution schema is represented in Figure 1.

## Methods

### Agile Development

The HSV patient registry development followed the agile framework defined by the UK Government Service Manual [32]. The alpha phase aims to develop the ideas formed in the previous discovery phase [3] and builds and tests prototype solutions. This involves examining the scope of the solution within the wider context of the users’ journey and developing an online prototype that is accessible for various users.

### Selection of a Database to Train and Test the Model

To develop and train the model, an initial data set that met the following requirements was needed:

- Open access, available without application or payment. This requirement is dictated by the fast iterative discovery approach that aims to maximize the speed and efficiency of the system development cycle
- A large number of patients included in the database; over 1000 rows are needed to provide sufficient data for machine learning model training and testing
- Clinically verified HSV diagnostic data
- Cross-referenced interviews and physical examination data
- An extensive list of demographics, lifestyle, and dietary variables
- High density of data points, meaning that most of the data fields are populated
- Verifiable data quality and reliability

The initial search was conducted via Google and patient registry lists using keywords related to human HSV patient databases and data sets. The data found on HSV patients consisted mostly of laboratory measurement data, which was not applicable for building a lifestyle-focused questionnaire. The US National Health and Nutrition Examination Survey (NHANES) was the only database identified that met all of the above requirements. Its data are in the public domain and can be used freely without obtaining copyright permission [33].

### The NHANES Database

The NHANES database was a major program conducted by the US National Centre for Health Statistics (under the umbrella of the Centers for Disease Control and Prevention). It provides high-density population data, gathered by high-quality standards, and details the methodology and data provenance [34], where all data are anonymized and are open access for statistical analysis. The data set used in this project was representative of the US population in 2015-2016 [35]. Moreover, the NHANES collected demographic information, enriched by detailed dietary, examination, and laboratory data, all linked with unique participant IDs. The survey is unique in that it combines interviews and physical examinations. The NHANES interview included demographic, socioeconomic, dietary, and health-related questions. The examination component consisted of medical, dental, and physiological measurements, as well as laboratory tests administered by highly trained medical personnel. All NHANES participants visited a physician. Dietary interviews, body measurements, blood sampling, and dental screening were included for all participants. Depending on the age of the participant, the rest of the examination included tests and procedures to assess the various aspects of health. HSV diagnosis was confirmed using diagnostic tests by physicians.

### HSV-1 and HSV-2 Data Sets

**Figure 2:**
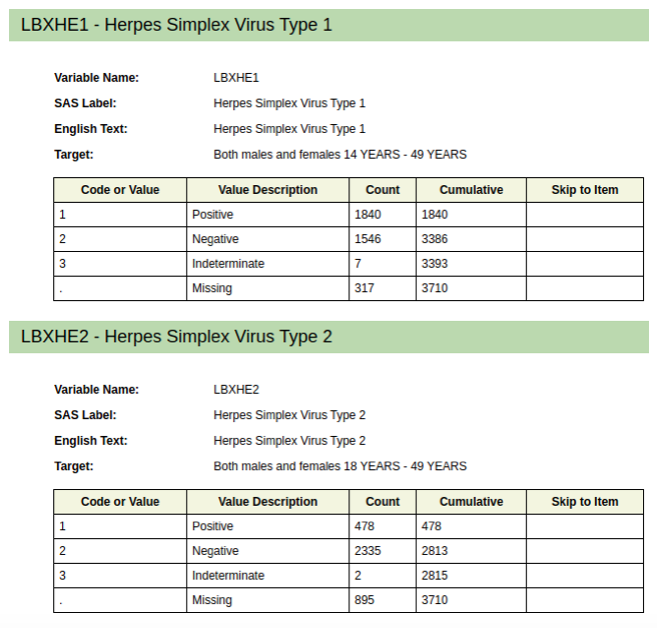
Demographics of the validation data set.

Two data sets—concerning HSV-1 (HSV type 1) and HSV-2—were taken from the NHANES database and used to train and validate the model. The demographics of the validation data sets for HSV-1 and HSV-2 can be found in Figure 2. Males and females, aged 14-49 years, were represented in the data set. The HSV-1 data set contains data from 3386 participants: 1840 confirmed positive for HSV-1 and 1546 confirmed negative. The HSV-2 data set contains data from 2813 participants: 478 confirmed positive for HSV-2 and 2335 confirmed negative. 

### Data Set Preparation

A complete data set with questionnaires and results for the period 2015-2016 is available on the National Center for Health Statistics website [34]. The overall initial list of questions is listed in Multimedia Appendix 1 and comprises over 600 questions. For the model, a smaller number of questions that contained sufficient cross-referenced data points to allow for analysis (n=150) was selected (Multimedia Appendix 2) to avoid the influence of missing data on the prediction results and to exclude some of the potentially sensitive questions. The value distribution for the HSV diagnosis confirmation (y) is 2335 (negative) and 478 (positive).

### Training and Testing Subsets

Using the data science method *train_test_split* from the *sklearn* Python library [36], the confirmed negative or positive cases reported in NHANES were randomly divided into two subdata sets for training and validation of the model with a ratio of 0.8 to 0.2. This ratio was chosen to keep the variance low and to leave enough data for training. The training data set was used to train the model, and the validation data set was used for accurate scoring. A threshold of 0.01 was experimentally defined to keep the list of questions as short as possible while maintaining good accuracy of the model. After checking feature importance values and determining that a *max_depth* value of 9 yields a threshold of feature importance less than 0.01, questions below the threshold were considered less relevant and were excluded.

### Cross-validation With a Grit Search

GridSearchCV was employed on the training data with cross-validation to tune the random forest parameters for variable selection size, several trees to generate (n_estimators=550), and maximum tree depth (max_depth=9). GridSearchCV accuracy was compared to that of RandomizedSearchCV (n_estimators=300, maximum tree depth [max_depth]=9), with a better performance of GridSearchCV on test accuracy—GridSearchCV train accuracy: 0.978, test accuracy: 0.957; RandomizedSearchCV train accuracy: 0.978, test accuracy: 0.954. Considering the higher speed of performance for RandomizedSearchCV, it may be more appropriate to use RandomizedSearchCV in the future production implementations of the model.

### Tools and Technology Stack for Model

A CART (classification and regression trees) random forest model was used to generate the main questionnaire. XGBoost approaches were also reviewed, but random forest performed better than XGBoost and with fewer complexities of implementation in production. Due to the high transparency and interpretability of CART models, a sequence of decision trees bagged into a random forest ensemble was chosen. The average decision tree plot, together with feature importance, was used to explore the full list of questions and define the shortest chain of interdependencies leading to HSV screening with the highest probability of accuracy.

The random forest ensemble was built from a sequence of decision trees using a bagging method [37]. Bagged decision tree ensembles are used to define entropy and information gain from previously selected features or discriminants [38]. Binary splitting on features with maximal informational gain leads to fewer nodes in the trees (ie, fewer relevant questions for diagnosing HSV). The model was designed to process the data in the following way:

1. The initial data set was divided into two subsets based on HSV type (1 or 2);
2. These data sets were randomly split by the data science method *train_test_split* from the sklearn Python library into a training set containing 80% of the samples and a validation set containing the remaining 20%;
3. The HSV-1 and HSV-2 training sets were processed by random forest classification estimators;
4. Accuracy on the training data sets was optimized by tuning the *max_depth* parameter (controls the total depth of the tree, that is, the number of binary splitting levels);
5. Accuracy was checked with validation subsets;
6. Values of *max_depth* gave the threshold of sufficient feature importance, and all questions below that level were excluded;
7. The final questions became the exhaustive list of features for the trained random forest classifier and used for the screening tool;
8. Given real-life data (questionnaire responses and clinical diagnosis verification), the model can improve its precision.

In this study, we designed and tested an algorithm that follows steps 1 to 7. Step 8, improvement of precision via integration within a live data collection system, is intended as a direction for future work.

### Illustration of the Random Forest Decision Tree

The decision tree (here bagged into a random forest ensemble) does sequences of binary splitting (splitting the sets of questions into two subgroups that produce the greatest distinction between positive and negative HSV diagnosis) until the resulting number of splittings is sufficient to explain the general tendencies of the data set (until the model has learned hidden patterns in data). Splittings are performed on the most informative feature, that is, the data feature having the highest information gain. In this particular case, a depth of 9 hierarchical levels of splitting was enough for the model to learn the connections between the data features and HSV-1/HSV-2 diagnosis.

The decision trees in Multimedia Appendices 3 and 4 show the final iteration of the random forest training process. The best results were achieved after 9 levels of branching, and further learning (splitting) brought no meaningful improvements in classification.

### Model Evaluation Metrics

The reduction in the number of questions needed to achieve high accuracy is an important success metric of the model. It can show the feasibility of using the model to reduce information entropy and encourage participants to complete the questionnaire. To validate the performance of the model, two key metrics were used: accuracy and recall score. Accuracy is the overall precision of the model in identifying HSV-positive patients from the questionnaire, whereas recall is a measure of the model’s capability to identify true positives.

Recall score is an important metric because it is preferable to identify a noninfected as being in the risk group than vice versa. The lowest possible score is 0 (0%), the highest is 1 (100% probability of true prediction). The recall was calculated using the equation:



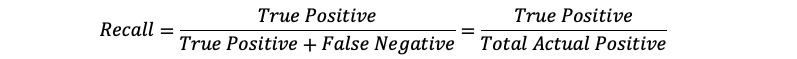



The resulting code can be found in a GitHub repository [39].

## Results

### Stage 1 Testing: NHANES Questions

The initial results of the random forest model computations are outlined in Table 1. As a result of the first stage of model development and testing, the number of questions was reduced from 150 to 62. The model selected a set of 62 questions that form shorter sequences for each user based on their age and gender. On average, a user would be asked 40 questions, with a minimum of 21 and a maximum of 62. It was estimated that it would take on average 25 minutes to answer all 150 questions depending on language and cognitive abilities, with the increased time required for sensitive questions. With only 40 questions, that would be reduced 3.75 times to less than 7 minutes. The final list of questions that could be presented to a user and the questionnaire flow can be found in Multimedia Appendix 5.

The confusion matrix for HSV-1 yielded 459 true positives, 81 true negatives, 8 false positives, and 15 false negatives.

**Table 1:** Stages 1 and 2 accuracy and recall scores.

Data set	Accuracy		Recall	
	Stage 1	Stage 2	Stage 1	Stage 2
HSV^a^ type 1	0.61	0.91	0.83	0.88
HSV type 2	0.83	0.96	0.90	0.98

^a^HSV: herpes simplex virus.

### Stage 2 Testing: NHANES Questions With Added Features

A lower accuracy score was found for the HSV-1 data set compared to the HSV-2 data set in stage 1. This meant that latent features inherited from NHANES were not strong enough to predict HSV-1 (Table 1). For example, the data set had limited symptom-related interview questions. Therefore, further research into HSV-1 and HSV-2 symptoms was conducted so that additional features could be introduced to the model and tested. A sample feature was added into the model based on literature suggesting that a significant proportion of people infected with HSV-1/HSV-2 virus types (up to 80%, depending on gender and virus type [40]) may experience more general symptoms like fever, muscle aches, and nausea. Therefore, a new question was added to the questionnaire for both HSV-1 and HSV-2 types: *Is your general feeling of discomfort or illness followed by one or more symptoms: fever, nausea, headaches, muscle pain, swollen lymph nodes, or malaise?* This additional feature was engineered for the data set, with a positive label in the 80% cases of the infected population. An additional question about symptoms with a high presence in HSV-infected people was introduced and improved the ability of the random forest model to train and test data predictions (Table 1).

Once the tool is in operation and is collecting real-world data (such that a significant number of participants answer the questionnaire and have their HSV status confirmed clinically), the model will gradually verify whether flu-like symptoms are a strong predictor of HSV. The model is intended to readjust the scoring method to exclude it as an important factor if this question turns out not to improve model accuracy.

## Discussion

### Principal Findings

This project has developed and successfully tested an optimization algorithm that minimizes the number of user-generated data points needed to accurately assess the risk of HSV-1 and HSV-2 infection. As a result of the implementation of the developed machine learning model, the algorithm was able to predict the HSV risk group attribution with high accuracy and recall scores, while the number of questions was reduced from 150 to 62. In the first stage of testing, the system was prototyped on the publicly available data of a small population of US citizens published in the NHANES database [3]. The second stage demonstrated how the same procedure could be repeated with additional variables (that are determined to be strongly linked to HSV infection) to achieve greater model accuracy. 

### Strengths and Limitations

A strength of the study is that it was conducted following the UK Government Service Manual for agile delivery [32] and was based on the principles established in a previously published discovery phase paper [3]. The project conducted fast technical prototyping to test the innovation’s assumptions, for example, that it is possible to use machine learning methods to improve direct patient data collection for sensitive topics. The results of this study will therefore provide inputs for further development and beta system design improvements.

One limitation of the project is that the model was tested and trained using precollected survey data. These data are limited in both size and dimensionality and were relatively sparsely populated. Access to larger data sets with more symptom-related information would have been beneficial and likely would have enabled greater model accuracy.

Another limitation is that the model has not yet been trained and tested on real user data in the context of a patient registry or online questionnaire. It was designed to be used in a machine learning system with a feedback loop that enables verification of the predicted HSV risk level with subsequent clinically confirmed diagnoses. Without real-world data, this function could not be tested in this study.

### Relation to Other Works

Many studies are applying decision trees and random forest machine learning models to patient databases to predict a variety of clinical risks [41-45]. However, there is only limited research into the application of machine learning algorithms to produce or deliver adaptive or optimized questionnaires [46,47]. While adaptive questionnaires do already have a place in clinical evaluations, these tend to be based on predetermined rules [20]. However, these papers do not consider the implications of these models for the development of patient registries to support ongoing data collection that will enable model self-improvement. 

### Future Directions

The ultimate aim of this project is to increase the quality and quantity of data collected and improve the probability of users disclosing sensitive information and volunteering for clinical trials. The next step toward achieving a patient registry that meets these aims is to integrate the model into an independent backend module and connect it with a question-outputting and answer-collecting front end. This will enable further improvement of the model and testing of its self-improvement capabilities on real-world data. Once real users start using the screening tool and the tool predictions are verified by a clinician through diagnostic tests, the model will self-learn and verify or discard assumptions about relationships between the questions and HSV status. The model could also be improved by integrating more user data from EHRs to generate more insights regarding what questions can be more predictive of an individual’s risk level.

Anonymization options, explicit permissions, and a standardized data schema that address the General Data Protection Regulation (GDPR), the Health Insurance Portability and Accountability Act (HIPAA), and the requirements of third-party interfaces (such as the Fast Healthcare Interoperability Resources [FHIR]) will be essential components for a platform based on the HSV screening tool. Some of the additional functionalities that could be considered for future research and system improvement are:

- Multiclass classification, where HSV-1 and HSV-2 data would be treated simultaneously: machine learning assembling would help researchers find additional patterns in the habits of patients with HSV in the generated response database;
- Adding descriptive user segmentation to the model: by defining the most recurring patient behavior and patients’ profile type, the probability of gathering more relevant data could be improved.

### Suggested Success Metrics for Future Work

According to the Gov.uk Service Design guidelines [32], the beta stage of the project will introduce and track key quantitative metrics of system performance. This tracking should include the following key metrics:

- Conversion rate: patients who visit the registry and proceed to start the questionnaire;
- Drop-off rate: patients who start the questionnaire and proceed to complete it;
- Retention rate: patients who complete the questionnaire and proceed to sign up to share personal data;
- Number of users who completed the screening questionnaire.

These metrics can be tracked by integrated database analytics and Google Analytics, which will also be important for accumulating user behavior data for future analytics and development.

### Intended User Journey

The platform will be tailored for the two main groups of users: researchers and members of the public (which includes patients with a confirmed diagnosis and users who may wish to participate in the study or have concerns regarding the risks of HSV). Members of the public will be able to get an assessment of their risk of having HSV and will be encouraged to engage with further data sharing to obtain further health insights and support research endeavors. The system will be designed so that after new users receive the results from the screening tool, they will be asked whether or not they want to register and be added to the database. If they decide not to be added, their responses will be saved anonymously in the database and can be used as an additional source of insights into populations that would not provide any data that required registration, which will help address the challenge of selection bias.

If they do opt to register, users would receive access to dashboards that help them track their health and provide personalized insights, news, and advice. Consent processes would allow users to agree to notifications, give consent for trial involvement and use of data, and communicate with a clinician. The user responses will be recorded in the database and the model will self-improve based on the incoming data as each HSV screening tool result is compared to a clinically confirmed diagnosis.

Researchers will be able to use the registry to complement clinical research and facilitate patient recruitment for clinical trials. Researchers will need to register, be verified by the system administrator, and log in to their account before accessing pseudo-anonymized data (ie, data where personally identifiable information has been removed, but links to the original personal data are preserved). They will be able to filter user data to identify subsets of users with the characteristics they are investigating, send invitations to trial and research groups, and create further questions to identify trial eligibility. Introducing other potentially relevant variables into the questionnaire and model would also provide a means for researchers to test other assumptions of HSV on real users. The various user flows identified for the platform are listed in Multimedia Appendix 6.

### Conclusion

This project successfully developed, trained, and tested a model to predict individuals’ risk of HSV-1 and HSV-2 infection based on an optimized set of questions on demographics, lifestyle, and symptoms. Using machine learning to determine the questions with the best predictive value means that patients need to answer fewer survey questions. This solution will improve the user-centricity of patient registry systems and will address the challenge of collecting relevant, high-quality data from patients with a stigmatized health condition such as HSV. In the context of using the model within a patient registry platform that would enable ongoing data collection and feedback process, the improved data collection process would lead to better research and patient outcomes by addressing the issues associated with data incompleteness, including selection bias and stigma. Future research and development of the system will use real-world data to improve the model, examine important anonymity, consent, interoperability, and data security concerns, and develop and evaluate a holistic patient registry system (with a front-end user interface and a back-end data architecture).

## Appendix

Multimedia Appendix 1 Initial set of questions from NHANES.

Multimedia Appendix 2 Questions selected to develop and train the model.

Multimedia Appendix 3 Random forest decision tree for HSV-1.

Multimedia Appendix 4 Random forest decision tree for HSV-2.

Multimedia Appendix 5 Flow diagram and final selected questions.

Multimedia Appendix 6 The schema of user journeys.

## Abbreviations

CART: classification and regression trees
EHR: electronic health record
FHIR: Fast Healthcare Interoperability Resources
GDPR: General Data Protection Regulation
HIPAA: Health Insurance Portability and Accountability Act
HSV: herpes simplex virus
HSV-1: herpes simplex virus, type 1
HSV-2: herpes simplex virus, type 2
NHANES: US National Health and Nutrition Examination Survey
