# Supplementary material for: Machine Learning for Risk Group Identification and User Data Collection in a Herpes Simplex Virus Patient Registry: Algorithm Development and Validation Study
Source: JMIRx Med. 2021 Jun 11;2(2):e25560. doi: 10.2196/25560 (PMC10414389; doi:10.2196/25560)
Supplement: Multimedia Appendix 1 [file xmed_v2i2e25560_app1.pdf]

1.1. Pregnant or lactating females.
1.2. Current or recurrent non-herpetic infection or any underlying condition that may predispose to infection or anyone who has been admitted to the hospital due to bacteremia, pneumonia or any other serious infection in the last 12 months.
1.3. History of malignancy (except patients with surgically cured basal cell or squamous cell skin cancers).
1.4. History of organ transplantation.
1.5. HIV-positive status determined by history at screening or known history of any other immunosuppressive disease.
1.6. Severe comorbidities (diabetes mellitus requiring insulin, CHF (NYHA class II or worse) MI, CVA or TIA within 3 months of screening visit, unstable angina pectoris, oxygen-dependent severe pulmonary disease.
1.7. Known hypersensitivity to Dimethyl sulfoxide (DMSO) - https://www.ncbi.nlm.nih.gov/pmc/articles/PMC116584/ .
1.8. Subject has an abnormal skin condition (e.g., acne, eczema, rosacea, psoriasis, albinism, or chronic vesiculo-bullous disorder) that occurs in the area ordinarily affected by herpes labialis.
1.9. Common triggers for reactivation are well known and include ultraviolet light, trauma, fatigue, stress, fever, inflammation, and menstruation.
1.10. Recent history of alcoholism or drug abuse.
1.11. Known allergy or intolerance to vaccine components [e.g., potassium glutamate (possible cross-reaction to monosodium glutamate), sucrose] or to a vaccine containing any of the same substances.
1.12. Known allergy or intolerance to silver nitrate or lidocaine.
1.13. Known allergy or intolerance to acyclovir or valacyclovir.
1.14. Any kidney disease, or renal insufficiency, defined as serum creatinine >1.5 mg/dl. Participants with a prior history of a single episode of pyelonephritis will be eligible.
1.15. Any other conditions that in the judgment of the investigator would preclude successful completion of the clinical trial.
1.16. Patients with evidence of chronic hepatitis B or C.
1.17. History of drug or alcohol abuse
1.18. Subjects with active liver disease.
1.19. History of immune disorders, HIV infection or currently receiving immunosuppressants
1.20. Known to have gastrointestinal malabsorption

5.1 Alcohol Use

1. Had at least 12 alcoholic drinks/1 yr?
2. Had at least 12 alcoholic drinks/lifetime?

3. In the past 12 months, how often did {you/SP} drink any type of alcoholic beverage? PROBE: How many days per week, per month, or per year did {you/SP} drink?
4. In the past 12 months, on those days that {you/SP} drank alcoholic beverages, on average, how many drinks did {you/he/she} have? By a drink, I mean a 12 oz. beer, a 5 oz. glass of wine, or one and a half ounces of liquor.)
5. In the past 12 months, on how many days did {you/SP} have {Display number} or more drinks of any alcoholic beverage? PROBE: How many days per week, per month, or per year did {you/SP} have {DISPLAY NUMBER} or more drinks in a single day?
6. Was there ever a time or times in {your/SP's} life when {you/he/she} drank {DISPLAY NUMBER} or more drinks of any kind of alcoholic beverage almost every day?
7. During the past 30 days, how many times did {you/SP} drink {DISPLAY NUMBER} or more drinks of any kind of alcohol in about two hours?

5.2 Audiometry

1. These next questions are about {your/SP's} hearing. Which statement best describes {your/SP's} hearing (without a hearing aid or other listening devices)? Would you say {your/his/her} hearing is excellent, good, that {you have/s/he has} a little trouble, moderate trouble, a lot of trouble, or {are you/is s/he} deaf?
2. These next questions refer to hearing without the use of a hearing aid or any other listening devices. If {you have/SP has} one ear that is better than the other, please answer the questions for the hearing in {your/SP's} better ear. Can {you/SP} usually hear and understand what a person says without seeing his or her face if that person whispers to {you/him/her} from across a quiet room?
3. Can {you/SP} usually hear and understand what a person says without seeing his or her face if that person talks in a normal voice to {you/him/her} from across a quiet room?
4. Can {you/SP} usually hear and understand what a person says without seeing his or her face if that person shouts to {you/him/her} from across a quiet room?
5. Can {you/SP} usually hear and understand what a person says without seeing his or her face if that person speaks loudly into {your/his/her} better ear?
6. How often {do you/does SP} find it difficult to follow a conversation if there is background noise, for example, when other people are talking, TV or radio is on, or children are playing? Would you say…
7. How often does {your/SP's} hearing cause {you/him/her}to feel frustrated when talking to members of {your/his/her} family or friends? Would you say…
8. {Have you/Has SP} ever had 3 or more ear infections? Please include ear infections {you/he/she} may have had when {you/he/she was} a child.
9. {Have you/Has SP} ever had a tube placed in {your/his/her} ear to drain the fluid from {your/his/her} ear?
10. A hearing test by a specialist is one that is done in a soundproof booth or room, or with headphones. Hearing specialists include audiologists, ear nose and throat doctors, and trained technicians or occupational nurses. When was the last time {you had/SP had} {your/his/her} hearing tested by a hearing specialist?

11. {Have you/Has SP} ever worn a hearing aid or cochlear implant?
12. Hearing aid or Cochlear implant?
13. In the past 12 months, how often {have you/has SP} worn a hearing aid?
14. {Have you/Has SP} ever used assistive listening devices (ALDs), such as FM systems, closed-captioned television, amplified telephone, relay services, or a sign-language interpreter?
15. In the past 12 months, {have you/has SP} been bothered by ringing, roaring, or buzzing in {your/his/her} ears or head that lasts for 5 minutes or more?
16. How long {have you/has SP} been bothered by this ringing, roaring, or buzzing in {your/his/her} ears or head?
17. In the past 12 months, how often {have you/has SP} had this ringing, roaring, or buzzing in {your/his/her} ears or head? Would you say…
18. {Are you/Is SP} bothered by ringing, roaring, or buzzing in {your/his/her} ears or head only after listening to loud sounds or loud music?
19. {Are you/Is SP} bothered by ringing, roaring, or buzzing in {your/his/her} ears or head when going to sleep?
20. How much of a problem is this ringing, roaring, or buzzing in {your/his/her} ears or head? Would you say…
21. This next question is about {your/SP's} use of firearms that {you/he/she} may have used for target shooting, hunting, for {your/his/her} job or in military service. {Have you/Has SP} ever used firearms for any reason?
22. How many total rounds {have you/has SP} ever fired?
23. How often {did you/did SP} wear hearing protection devices (ear plugs, ear muffs) when shooting firearms?
24. These next questions are about noise exposure at work. First we are going to ask about loud noise. Loud means so loud that {you/s/he} must speak in a raised voice to be heard by someone three feet away when not using hearing protection. After that we will ask about very loud noise. Very loud noise is noise that is so loud {you have/he/she has} to shout in order to be understood by someone standing 3 feet away from {you/him/her} when not using hearing protection. {Have you/Has SP} ever had a job, or combination of jobs where {you were/s/he was} exposed to loud sounds or noise for 4 or more hours a day, several days a week? (Loud means so loud that {you/s/he} must speak in a raised voice to be heard.)
25. For how many months or years {have you/has SP} been exposed at work to loud sounds or noise for 4 or more hours a day, several days a week?
26. In {your/SP's} work {were you/was he/she} exposed to very loud noise? (Very loud noise is noise that is so loud {you have/he/she has} to shout in order to be understood by someone standing 3 feet away from {you/him/her}.)
27. How long exposed to very loud noise?
28. Outside of a job, {have you/has SP} ever been exposed to very loud noise or music for 10 or more hours a week? This is noise so loud that {you have/s/he has} to shout to be understood or heard 3 feet away. Examples are noise from power tools, lawn mowers, farm machinery, cars, trucks, motorcycles, motor boats or loud music.

29. In the past 12 months, how often {did you/did SP} wear hearing protection devices (ear plugs, ear muffs) when exposed to very loud sounds or noise outside of work? {Do not include the noise from firearms we already talked about.}

5.3 Blood Pressure & Cholesterol

1. {Have you/Has SP} ever been told by a doctor or other health professional that {you/s/he} had hypertension, also called high blood pressure?
2. {Were you/Was SP} told on 2 or more different visits that {you/s/he} had hypertension, also called high blood pressure?
3. How old {were you/was SP} when {you/he/she was} first told that {you/he/she} had hypertension or high blood pressure?
4. Because of {your/SP's} (high blood pressure/hypertension), {have you/has s/he} ever been told to . . . take prescribed medicine?
5. (Are you/Is SP) now taking prescribed medicine
6. {Have you/Has SP} ever been told by a doctor or other health professional that {your/his/her} blood cholesterol level was high?
7. {Have you/Has SP} ever had {your/his/her} blood cholesterol checked?
8. About how long has it been since {you/SP} last had {your/his/her} blood cholesterol checked? Has it been…
9. [To lower (your/his/her) blood cholesterol, (have/has) (you/SP) ever been told by a doctor or other health professional]... to take prescribed medicine?
10. (Are you/Is SP) now following this advice to take prescribed medicine?

5.4 Cardiovascular Health

1. {Have you/Has SP} ever had any pain or discomfort in {your/her/his} chest?
2. {Do you/Does she/Does he} get it when {you/she/he} walk uphill or hurry?
3. {Do you/Does she/Does he} get it when {you/she/he} walk at an ordinary pace on level ground?
4. What {do you/does she/he} do if {you/she/he} get it while {you/she/he} are walking? {Do you/Does she/Does he} stop or slow down or continue at the same pace?
5. If {you/she/he} stand still, what happens to it? Is the pain or discomfort relieved or not relieved?
6. How soon is the pain relieved? Would you say…
7. Pain in right arm
8. Pain in right chest
9. Pain in neck
10. Pain in upper sternum
11. Pain in lower sternum
12. Pain in left chest
13. Pain in left arm
14. Pain in epigastric area

15. Have {you/she/he} ever had a severe pain across the front of {your/her/his} chest lasting for half an hour or more?
16. {Have you/Has SP} had shortness of breath either when hurrying on the level or walking up a slight hill?

5.5 Consumer Behavior

1. The next questions are about how much money {your family spends/you spend} on food. First I'll ask you about money spent at supermarkets or grocery stores. Then we will talk about money spent at other types of stores. When you answer these questions, please do not include money spent on alcoholic beverages. During the past 30 days, how much money {did your family/did you} spend at supermarkets or grocery stores? Please include purchases made with food stamps. (You can tell me per week or per month.)
2. About how much money was spent on nonfood items? (You can tell me per week or per month.)
3. About how much money {did your family/did you} spend on food at these types of stores? Please do not include money you have already told me about. (You can tell me per week or per month.)
4. During the past 30 days, how much money {did your family/did you} spend on eating out? Please include money spent in cafeterias at work or at school or on vending machines, for all family members. (You can tell me per week or per month.)
5. During the past 30 days, how much money {did your family/did you} spend on food carried out or delivered? Please do not include money you have already told me about. (You can tell me per week or per month.)

5.6 Current Health Status

1. Next I have some general questions about {your/SP's} health. Would you say {your/SP's} health in general is . . .
2. Did {you/SP} have a head cold or chest cold that started during those 30 days?
3. Did {you/SP} have a stomach or intestinal illness with vomiting or diarrhea that started during those 30 days?
4. Did {you/SP} have flu, pneumonia, or ear infections that started during those 30 days?
5. During the past 12 months, that is, since (DISPLAY CURRENT MONTH, DISPLAY LAST YEAR), (have you/has SP) donated blood?
6. How long ago was {your/SP's} last blood donation?
7. Except for tests {you/SP} may have had as part of blood donations, {have you/has he/has she} ever had {your/his/her} blood tested for the AIDS virus infection?
8. Source of Health Status Data

5.7 Dermatology

1. If after several months of not being in the sun, {you/SP} then went out in the sun without sunscreen or protective clothing for a half hour, which one of these would happen to {your/his/her} skin?
2. When {you go/SP goes} outside on a very sunny day, for more than one hour, how often {do you/does SP} Stay in the shade?
3. Wear a long sleeved shirt? Would you say
4. Use sunscreen? Would you say
5. How many times in the past year {have you/has SP} had a sunburn?
6. The next questions ask about the time you spent outdoors during the past 30 days. By outdoors, I mean outside and not under any shade. How much time did you usually spend outdoors between 9 in the morning and 5 in the afternoon on the days that you worked or went to school?
7. During the past 30 days, how much time did you usually spend outdoors between 9 in the morning and 5 in the afternoon on the days when you were not working or going to school?

5.8 Diabetes

1. The next questions are about specific medical conditions. {Other than during pregnancy, {have you/has SP}/{Have you/Has SP}} ever been told by a doctor or health professional that {you have/{he/she/SP} has} diabetes or sugar diabetes?
2. How old {wasP/were you} when a doctor or other health professional first told {you/him/her} that {you/he/she} had diabetes or sugar diabetes?
3. {Have you/Has SP} ever been told by a doctor or other health professional that {you have/SP has} any of the following: prediabetes, impaired fasting glucose, impaired glucose tolerance, borderline diabetes or that {your/her/his} blood sugar is higher than normal but not high enough to be called diabetes or sugar diabetes?
4. {Have you/Has SP} ever been told by a doctor or other health professional that {you have/s/he has} health conditions or a medical or family history that increases {your/his/her} risk for diabetes?
5. {Do you/Does SP} feel {you/he/she} could be at risk for diabetes or prediabetes?
6. Why {Do you/Does SP} think {you are/he is/she is} at risk for diabetes or prediabetes? [Anything else?]
7. Why {Do you/Does SP} think {you are/he is/she is} at risk for diabetes or prediabetes? [Anything else?]
8. {Have you/Has SP} had a blood test for high blood sugar or diabetes within the past three years?
9. {Is SP/Are you} now taking insulin
10. For how long {have you/has SP} been taking insulin?

11. {Is SP/Are you} now taking diabetic pills to lower {{his/her}/your} blood sugar? These are sometimes called oral agents or oral hypoglycemic agents.
12. When was the last time {you/SP} saw a diabetes nurse educator or dietitian or nutritionist for {your/his/her} diabetes? Do not include doctors or other health professionals.
13. Is there one doctor or other health professional {you usually see/SP usually sees} for {your/his/her} diabetes? Do not include specialists to whom {you have/SP has} been referred such as diabetes educators, dieticians or foot and eye doctors.
14. How many times {have you/has SP} seen this doctor or other health professional in the past 12 months?
15. How often {do you check your/does SP check his/her} blood for glucose or sugar? Include times when checked by a family member or friend, but do not include times when checked by a doctor or other health professional.
16. How often {do you check your/does SP check his/her} blood for glucose or sugar? Include times when checked by a family member or friend, but do not include times when checked by a doctor or other health professional.
17. Glycosylated (GLY-KOH-SIH-LAY-TED) hemoglobin or the "A one C" test measures your average blood sugar for the past 3 months, and usually ranges between 5.0 and 13.9. During the past 12 months, has a doctor or other health professional checked {your/SP's} glycosylated hemoglobin or "A one C"?
18. What was {your/SP's} last "A one C" level?
19. What does {your/SP's} doctor or other health professional say {your/his/her} "A one C" level should be? (Pick the lowest level recommended by your healthcare professional.)
20. Blood pressure is usually given as one number over another. What was {your/SP's} most recent blood pressure in numbers?
21. Blood pressure is usually given as one number over another. What was {your/SP's} most recent blood pressure in numbers?
22. What does Dr say SBP should be
23. What does Dr say DBP should be
24. One part of total serum cholesterol in {your/SP's} blood is a bad cholesterol, called LDL,which builds up and clogs {your/his/her} arteries. What was {your/his/her} most recent LDL cholesterol number?
25. What does {your/SP's} doctor or other health professional say {your/his/her} LDL cholesterol should be?
26. During the past 12 months, about how many times has a doctor or other health professional checked {your/SP's} feet for any sores or irritations?
27. How often {do you check your feet/does SP check (his/her) feet} for sores or irritations? Include times when checked by a family member or friend, but do not include times when checked by a doctor or other health professional.
28. When was the last time {you/SP} had an eye exam in which the pupils were dilated? This would have made {you/SP} temporarily sensitive to bright light.
29. Has a doctor ever told {you/SP} that diabetes has affected {your/his/her} eyes or that {you/s/he} had retinopathy (ret-in-op-ath-ee)?

5.9 Diet Behavior & Nutrition

1. Now I'm going to ask you some general questions about {SP's} eating habits. Was {SP} ever breastfed or fed breast milk?
2. How old was {SP} when {he/she} completely stopped breastfeeding or being fed breast milk?
3. How old was {SP} when {he/she} was first fed formula?
4. How old was {SP} when {he/she} completely stopped drinking formula?
5. This next question is about the first thing that {SP} was given other than breast milk or formula. Please include juice, cow's milk, sugar water, baby food, or anything else that {SP} might have been given, even water. How old was {SP} when {he/she} was first fed anything other than breast milk or formula? (Days)
6. How old was {SP} when {he/she} was first fed milk?
7. What type of milk was {SP} first fed? Was it .
8. Next I have some questions about {your/SP?s} eating habits. In general, how healthy is {your/his/her} overall diet? Would you say . . .
9. Now I'm going to ask a few questions about milk products. Do not include their use in cooking. In the past 30 days, how often did {you/SP} have milk to drink or on {your/his/her} cereal? Please include chocolate and other flavored milks as well as hot cocoa made with milk. Do not count small amounts of milk added to coffee or tea. Would you say…
10. What type of milk do you drink?
11. The next question is about regular milk use. A regular milk drinker is someone who uses any type of milk at least 5 times a week. Using this definition, which statement best describes {you/SP}?...
12. Now, I'm going to ask you how often {you/SP} drank milk at different times in {your/his/her} life. How often did {you/SP} drink any type of milk, including milk added to cereal when {you were/s/he was} a child between the ages of 5 and 12 years old? Would you say…
13. Now, I'm going to ask you how often {you/SP} drank milk at different times in {your/his/her} life. How often did {you/SP} drink any type of milk, including milk added to cereal when {you were/s/he was} a teenager between the ages of 13 and 17 years old? Would you say…
14. Now, I'm going to ask you how often {you/SP} drank milk at different times in {your/his/her} life. How often did {you/SP} drink any type of milk, including milk added to cereal when {you were/s/he was} a young adult between the ages of 18 and 35 years old? Would you say..
15. The next questions are about meals provided by community or government programs. In the past 12 months, did {you/SP} receive any meals delivered to {your/his/her} home from community programs, "Meals on Wheels", or any other programs?
16. In the past 12 months, did {you/SP} go to a community program or senior center to eat prepared meals?

17. During the school year, {do you/does SP} attend kindergarten, grade school, junior or high school?
18. Does {your/SP's} school serve school lunches? These are complete lunches that cost the same every day.
19. During the school year, about how many times a week {do you/does SP} usually get a complete school lunch?
20. {Do you/Does SP} get these lunches free, at a reduced price, or {do you/does he/she} pay full price?
21. Does {your/SP's} school serve a complete breakfast that costs the same every day?
22. During the school year, about how many times a week {do you/does SP} usually get a complete breakfast at school?
23. {Do you/Does SP} get these breakfasts free, at a reduced price, or {do you/does he/she} pay full price?
24. (Do you/Does SP) get a free or reduced price meal at any summer program (he/she) attends?
25. Next I'm going to ask you about meals. By meal, breakfast, lunch and dinner. During the past 7 days, how many meals {did you/did SP} get that were prepared away from home in places such as restaurants, fast food places, food stands, grocery stores, or from vending machines? {Please do not include meals provided as part of the school lunch or school breakfast./Please do not include meals provided as part of the community programs you reported earlier.}
26. How many of those meals {did you/did SP} get from a fast-food restaurant or pizza place?
27. Some grocery stores sell "ready to eat" foods such as salads, soups, chicken, sandwiches and cooked vegetables in their salad bars and deli counters. During the past 30 days, how often did {you/SP} eat "ready to eat" foods from the grocery store? Please do not include sliced meat or cheese you buy for sandwiches and frozen or canned foods.
28. During the past 30 days, how often did you {SP} eat frozen meals or frozen pizzas? Here are some examples of frozen meals and frozen pizzas.
29. Next I'm going to ask a few questions about the nutritional guidelines recommended for Americans by the federal government. {Have you/Has SP} heard of My Plate?
30. {Have you/Has SP} looked up the MyPlate plan on the internet?
31. {Have you/Has SP} tried to follow the recommendations in the MyPlate plan?
32. {I'll tell you when you will need it.} For the first few questions, please answer yes or no. In the past 12 months, did you buy food from fast food or pizza places? SP interview version: In the past 12 months, did {you/SP} buy food from fast food or pizza places?
33. The last time you ate out or bought food at a fast-food restaurant or pizza place, did you see nutrition or health information about any foods on the menu? SP interview version: The last time when {you/SP} ate out or bought food at a fast-food restaurant or pizza place, did {you/he/she} see nutrition or health information about any foods on the menu?
34. Did you use the information in deciding which foods to buy? SP interview version: Did {you/SP} use the information in deciding which foods to buy?

35. {Please open your hand card booklet and turn to hand card 1 to answer the next question.} If nutrition or health information were readily available in fast food or pizza places, would you use it often, sometimes, rarely, or never, in deciding what to order? SP interview version: If nutrition or health information were readily available in fast food or pizza places, would {you/SP} use it often, sometimes, rarely, or never, in deciding what to order?
36. For the following questions, please answer yes or no.] In the past 12 months, did you eat at a restaurant with waiter or waitress service? SP interview version: In the past 12 months, did {you/SP} eat at a restaurant with waiter or waitress service?
37. Think about the last time {you/SP} ate at a restaurant with a waiter or waitress. Is it a chain-restaurant?
38. The last time you ate at a restaurant with a waiter or waitress, did you see nutrition or health information about any foods on the menu? SP interview version: Did {you/SP} see nutrition or health information about any foods on the menu?
39. Did you use the information in deciding which foods to buy? SP interview version: Did {you/SP} use the information in deciding which foods to buy?
40. {Please look at hand card 1 [again].} If nutrition information were readily available in restaurants with a waiter or waitress, would you use it often, sometimes, rarely, or never, in deciding what to order? SP interview version: If nutrition or health information were readily available in restaurants with a waiter or waitress, would {you/SP} use it often, sometimes, rarely, or never, in deciding what to order?

5.10 Disability

1. With this next set of questions, we want to learn about people who have physical, mental, or emotional conditions that cause serious difficulty with their daily activities. Though different, these questions may sound similar to ones I asked earlier. {Are you/Is SP} deaf or {do you/does he/she} have serious difficulty hearing?
2. {Are you/Is SP} blind or {do you/does he/she} have serious difficulty seeing even when wearing glasses?
3. Because of a physical, mental, or emotional condition, {do you/does he/she} have serious difficulty concentrating, remembering, or making decisions?
4. {Do you/Does SP} have serious difficulty walking or climbing stairs?
5. {Do you/Does SP} have difficulty dressing or bathing?
6. Because of a physical, mental, or emotional condition, {do you/does he/she} have difficulty doing errands alone such as visiting a doctor's office or shopping?
7. How often do you feel worried, nervous or anxious? Would you say daily, weekly, monthly, a few times a year, or never?
8. Do you take medication for these feelings?
9. Thinking about the last time you felt worried, nervous or anxious, how would you describe the level of these feelings? Would you say a little, a lot, or somewhere in between?

10. How often do you feel depressed? Would you say daily, weekly, monthly, a few times a year, or never?
11. Do you take medication for depression?
12. Thinking about the last time you felt depressed, how depressed did you feel? Would you say a little, a lot, or somewhere in between?

5.11 Drug Use

1. The following questions ask about the use of drugs not prescribed by a doctor. Please remember that your answers to these questions are strictly confidential. The first questions are about marijuana and hashish. Marijuana is also called pot or grass. Marijuana is usually smoked, either in cigarettes called joints, or in a pipe. It is sometimes cooked in food. Hashish is a form of marijuana that is also called 'hash.' It is usually smoked in a pipe. Another form of hashish is hash oil. Have you ever, even once, used marijuana or hashish?
2. How old were you the first time you used marijuana or hashish?
3. Have you ever smoked marijuana or hashish at least once a month for more than one year?
4. How old were you when you started smoking marijuana or hashish at least once a month for one year?
5. How long has it been since you last smoked marijuana or hashish at least once a month for one year?
6. How long has it been since you last smoked marijuana or hashish at least once a month for one year?
7. During the time that you smoked marijuana or hashish, how often would you usually use it?
8. During the time that you smoked marijuana or hashish, how many joints or pipes would you usually smoke in a day?
9. How long has it been since you last used marijuana or hashish?
10. During the past 30 days, on how many days did you use marijuana or hashish?
11. Have you ever used cocaine, crack cocaine, heroin, or methamphetamine?
12. The following questions are about cocaine, including all the different forms of cocaine such as powder, 'crack', 'free base', and coca paste. Have you ever, even once, used cocaine, in any form?
13. How old were you the first time you used cocaine, in any form?
14. How long has it been since you last used cocaine, in any form?
15. During your life, altogether, how many times have you used cocaine, in any form?
16. During the past 30 days, on how many days did you use cocaine, in any form?
17. The following questions are about heroin. Have you ever, even once, used heroin?
18. How old were you the first time you used heroin?
19. How long has it been since you last used heroin?
20. During the past 30 days, on how many days did you use heroin?

21. The following questions are about methamphetamine, also known as crank, crystal, ice or speed. Have you ever, even once, used methamphetamine?
22. How old were you the first time you used methamphetamine?
23. How long has it been since you last used methamphetamine?
24. During your life, altogether how many times have you used methamphetamine?
25. During the past 30 days, on how many days did you use methamphetamine?
26. The following questions are about the different ways that certain drugs can be used. Have you ever, even once, used a needle to inject a drug not prescribed by a doctor?
27. Which of the following drugs have you injected using a needle?
28. How old were you when you first used a needle to inject any drug not prescribed by a doctor?
29. How long has it been since you last used a needle to inject a drug not prescribed by a doctor?
30. During your life, altogether, how many times have you injected drugs not prescribed by a doctor?
31. Think about the period of your life when you injected drugs the most often. How often did you inject then?
32. Have you ever been in a drug treatment or drug rehabilitation program?

5.12 Early Childhood

1. First, I have some questions about {SP NAME's} birth. How old was {SP NAME's} biological mother when {s/he} was born?
2. Did {SP NAME's} biological mother smoke at any time while she was pregnant with {him/her}?
3. How much did {SP NAME} weigh at birth?
4. Weight more/less than 5.5 lbs
5. Weight more/less than 9.0 lbs
6. How do you consider {SP} weight?
7. Has a doctor or health professional ever told you that {SP} was overweight?
8. Are you doing anything to help {SP} control {his/her} weight?

5.13 Health Insurance

1. The (first/next) questions are about health insurance. {Are you/Is SP} covered by health insurance or some other kind of health care plan? [Include health insurance obtained through employment or purchased directly as well as government programs like Medicare and Medicaid that provide medical care or help pay medical bills.]
2. {Are you/Is SP} covered by private insurance?
3. {Are you/Is SP} covered by Medicare?
4. {Are you/Is SP} covered by Medi-Gap?
5. {Are you/Is SP} covered by Medicaid?
6. {Are you/Is SP} covered by SCHIP (State Children's Health Insurance Program)?

7. {Are you/Is SP} covered by military health plan (Tricare/VA/Champ-VA)?
8. {Are you/Is SP} covered by Indian Health Service?
9. {Are you/Is SP} covered by state-sponsored health plan?
10. {Are you/Is SP} covered by other government insurance?
11. {Are you/Is SP} covered by any single service plan?
12. No coverage of any type.
13. {Do you/Does SP} have Medicare?
14. Insurance card available or not.
15. {Does this plan/Do any of these plans} cover any part of the cost of prescriptions?
16. In the past 12 months, was there any time when {you/SP} did not have any health insurance coverage?

5.14 Hepatitis

1. Has a doctor or other health professional ever told {you/SP} that {you have/s/he/SP has} Hepatitis B? (Hepatitis is a form of liver disease. Hepatitis B is an infection of the liver from the Hepatitis B virus (HBV).)
2. Please look at the drugs on this card that are prescribed for Hepatitis B. {Were you/Was/s/he/SP} ever prescribed any medicine to treat Hepatitis B?
3. Has a doctor or other health professional ever told {you/SP} that {you have/s/he/SP has} Hepatitis C? (Hepatitis is a form of liver disease. Hepatitis C is an infection of the liver from the Hepatitis C virus (HCV).)
4. Please look at the drugs on this card that are prescribed for Hepatitis C. {Were you/ Was/s/he/SP} ever prescribed any medicine to treat Hepatitis C?

5.15 Hospital Utilization & Access to Care

1. {First/Next} I have some general questions about {your/SP's} health. Would you say {your/SP's} health in general is . . .
2. Compared with 12 months ago, would you say {your/SP's} health is now . . .
3. Is there a place that {you/SP} usually {go/goes} when {you are/he/she is} sick or {you/s/he} need{s} advice about {your/his/her} health?
4. {What kind of place is it - a clinic, doctor's office, emergency room, or some other place?} {What kind of place {do you/does SP} go to most often - a clinic, doctor's office, emergency room, or some other place?}
5. {During the past 12 months, how/How} many times {have you/has SP} seen a doctor or other health care professional about {your/his/her} health at a doctor's office, a clinic or some other place? Do not include times {you were/s/he was} hospitalized overnight, visits to hospital emergency rooms, home visits or telephone calls.
6. About how long has it been since {you/SP} last saw or talked to a doctor or other health care professional about {your/his/her} health? Include doctors seen while {you were} {he/she was} a patient in a hospital. Has it been . . .

7. {During the past 12 months, were you/{was} SP} a patient in a hospital overnight? Do not include an overnight stay in the emergency room.
8. How many different times did {you/SP} stay in any hospital overnight or longer {during the past 12 months}? (Do not count the total number of nights, just total number of hospital admissions for stays which lasted 1 or more nights.)
9. During the past 12 months, that is since {DISPLAY CURRENT MONTH} of {DISPLAY LAST YEAR}, {have you/has SP} seen or talked to a mental health professional such as a psychologist, psychiatrist, psychiatric nurse or clinical social worker about {your/his/her} health?

5.16 Housing Characteristics

1. How many rooms are in this home? Count the kitchen but not the bathroom.
2. Is this {mobile home/house/apartment} owned, being bought, rented, or occupied by some other arrangement by {you/you or someone else in your family}?

5.17 Immunization

1. Human Papillomavirus (HPV) vaccine is given to prevent cervical cancer in girls and women. The HPV vaccines available are called Cervarix, Gardasil or Gardasil 9. It is given in 3 separate doses over a 6 month period. {Have you/Has SP} ever received one or more doses of the HPV vaccine?
2. Human Papillomavirus (HPV) vaccine is given to prevent HPV infection and genital warts in boys and men. It is given in 3 separate doses over a 6 month period. {Have you/Has SP} ever received one or more doses of the HPV vaccine? (The brand name for the vaccine is Gardasil.)
3. Which of the HPV vaccines did {you/SP} receive, Cervarix or Gardasil?
4. How old {were you/was SP} when {you/SP} received your first dose of {Cervarix/Gardasil/Gardasil 9/Gardasil or Gardasil 9/the vaccine}?
5. How many doses of {Cervarix/Gardasil/Gardasil or Gardasil 9/the vaccine} {have you/has SP} received?

5.18 Income

1. The next questions are about {your/your combined family} income. When answering these questions, please remember that by {"income/combined family income"}, I mean {your income/your income plus the income of {NAMES OF OTHER NHANES FAMILY MEMBERS} for {LAST CALENDAR YEAR}. Did {you/you and OTHER NHANES FAMILY MEMBERS 16+} receive income in {LAST CALENDAR YEAR} from wages and salaries? [Did {you/you or OTHER FAMILY MEMBERS 16+} get paid for work in {LAST CALENDAR YEAR}.]

2. Did {you/you or any family members 16 and older} receive income in {LAST CALENDAR YEAR} from self-employment including business and farm income? [Self-employment means you worked for yourself.]
3. When answering the next questions about different kinds of income members of your family might have received in {LAST CALENDAR YEAR}, please consider that we also want to know about family members less than 16 years old. Did {you/you or any family members living here, that is: you or NAME(S) OF OTHER NHANES FAMILY MEMBERS} receive income in {LAST CALENDAR YEAR} from Social Security or Railroad Retirement?
4. Did {you/you or any family members living here} receive any disability pension [other than Social Security or Railroad Retirement] in {LAST CALENDAR YEAR}?
5. Did {you/you or any family members living here} receive retirement or survivor pension [other than Social Security or Railroad Retirement or disability pension] in {LAST CALENDAR YEAR}?
6. Did {you/you or any family members living here} receive Supplemental Security Income [SSI] in {LAST CALENDAR YEAR}?
7. Did {you/you or any family members living here} receive any cash assistance from a state or county welfare program such as {DISPLAY SPECIFIC STATE PROGRAMS} in {LAST CALENDAR YEAR}?
8. Did {you/you or any family members living here} receive interest from savings or other bank accounts or income from dividends received from stocks or mutual funds or net rental income from property, royalties, estates, or trusts in {LAST CALENDAR YEAR}? Did {you/you or any family members living here} receive income in {LAST CALENDAR YEAR} from child support, alimony, contributions from family or others, VA payments, worker's compensation, or unemployment compensation?
9. Monthly family income (reported as a range value in dollars).
10. Family monthly poverty level index, a ratio of monthly family income to the HHS poverty guidelines specific to family size.
11. Family monthly poverty level index categories.
12. Do {you/NAMES OF OTHER FAMILY/you and NAMES OF FAMILY MEMBERS} have more than $20,000 in savings at this time? Please include money in your checking accounts.
13. Total savings or cash assets at this time for {you/NAMES OF OTHER FAMILY/your family}.
14. Please look at this card. How do {you/you or anyone who lives in the household} usually get to the store (or stores) where you do most of your grocery shopping?

5.19 Kidney Conditions - Urology

1. {Have you/Has SP} ever been told by a doctor or other health professional that {you/s/he} had weak or failing kidneys? Do not include kidney stones, bladder infections, or incontinence.

2. In the past 12 months, {have you/has SP} received dialysis (either hemodialysis or peritoneal dialysis)?
3. {Have you/Has SP} ever had kidney stones?
4. In the past 12 months {have you/has SP} passed a kidney stone?
5. Many people have leakage of urine. The next few questions ask about urine leakage. How often {do you/does SP} have urinary leakage? Would {you/s/he} say . . .
6. How much urine {do you/does SP} lose each time? Would {you/s/he} say . . .
7. During the past 12 months, {have you/has SP} leaked or lost control of even a small amount of urine with an activity like coughing, lifting or exercise?
8. How frequently does this occur? Would {you/s/he} say this occurs
9. During the past 12 months, {have you/has SP} leaked or lost control of even a small amount of urine with an urge or pressure to urinate and {you/he/she} couldn't get to the toilet fast enough?`
10. How frequently does this occur? Would {you/s/he} say this occurs.
11. During the past 12 months, {have you/has SP} leaked or lost control of even a small amount of urine without an activity like coughing, lifting, or exercise, or an urge to urinate?
12. How frequently does this occur? Would {you/s/he} say this occurs
13. During the past 12 months, how much did {your/her/his} leakage of urine bother {you/her/him}?
14. During the past 12 months, how much did {your/her/his} leakage of urine affect {your/her/his} day-to-day activities?
15. During the past 30 days, how many times per night did {you/SP} most typically get up to urinate, from the time {you/s/he} went to bed at night until the time {you/he/she} got up in the morning. Would {you/s/he} say

5.20 Medical Conditions

1. The following questions are about different medical conditions. Has a doctor or other health professional ever told {you/SP} that {you have/s/he/SP has} asthma (az-ma)?
2. How old {were you/was SP} when {you were/s/he was} first told {you/he/she} had asthma (az-ma)?
3. {Do you/Does SP} still have asthma (az-ma)?
4. During the past 12 months, {have you/has SP} had an episode of asthma (az-ma) or an asthma attack?
5. [During the past 12 months], {have you/has SP} had to visit an emergency room or urgent care center because of asthma
6. During the past 12 months, {have you/has SP} had an episode of hay fever?
7. During the past 3 months, {have you/has SP} been on treatment for anemia (a-nee-me-a), sometimes called "tired blood" or "low blood"? [Include diet, iron pills, iron shots, transfusions as treatment.]
8. Has a doctor or other health professional ever told {you/SP} that {you/s/he/SP was} overweight?

9. {Have you/Has SP} ever received a blood transfusion?
10. In what year did {you/SP} receive {your/his/her} first transfusion?
11. Have {SP's} periods or menstrual (men-steal) cycles started yet?
12. How old was {SP} when she had {her} first menstrual period?
13. Has a doctor or other health professional ever told {you/SP} that {you/s/he} . . .had arthritis (ar-thry-tis)?
14. How old {were you/was SP} when {you were/s/he was} first told {you/s/he} . . . had arthritis?
15. Which type of arthritis was it?
16. Has a doctor or other health professional ever told {you/SP} that {you/s/he} . . .had gout?
17. How old {were you/was SP} when {you were/s/he was} first told {you/s/he} . . .had gout?
18. Has a doctor or other health professional ever told {you/SP} that {you/s/he} . . .had congestive heart failure?
19. How old {were you/was SP} when {you were/s/he was} first told {you/s/he} . . .had congestive heart failure?
20. Has a doctor or other health professional ever told {you/SP} that {you/s/he} . . .had coronary heart disease?
21. Has a doctor or other health professional ever told {you/SP} that {you/s/he} . . .had angina (an-gi-na), also called angina pectoris?
22. How old {were you/was SP} when {you were/s/he was} first told {you/s/he} . . .had angina, also called angina pectoris?
23. Has a doctor or other health professional ever told {you/SP} that {you/s/he} . . .had a heart attack (also called myocardial infarction
24. How old {were you/was SP} when {you were/s/he was} first told {you/s/he} . . .had a heart attack (also called myocardial infarction)?
25. Has a doctor or other health professional ever told {you/SP} that {you/s/he} . . .had a stroke?
26. How old {were you/was SP} when {you were/s/he was} first told {you/s/he} . . .had a stroke?
27. Has a doctor or other health professional ever told {you/SP} that {you/s/he} . . .had emphysema
28. How old {were you/was SP} when {you were/s/he was} first told {you/s/he} . . .had emphysema?
29. Has a doctor or other health professional ever told {you/SP} that {you/s/he} . . .had another thyroid (thigh-roid) problem?
30. {Do you/Does SP} still . . . have another thyroid problem?
31. How old {were you/was SP} when {you were/s/he was} first told {you/s/he} . . .had another thyroid problem?
32. Has a doctor or other health professional ever told {you/SP} that {you/s/he} . . .had chronic bronchitis?
33. {Do you/Does SP} still . . . have chronic bronchitis?
34. How old {were you/was SP} when {you were/s/he was} first told {you/s/he} . . .had chronic bronchitis?

35. Has a doctor or other health professional ever told {you/SP} that {you/s/he} . . .had any kind of liver condition?
36. {Do you/Does SP} still . . . have any kind of liver condition?
37. How old {were you/was SP} when {you were/s/he was} first told {you/s/he} . . .had any kind of liver condition?
38. Has a doctor or other health professional ever told {you/SP} that {you/s/he} . . .had COPD?
39. Has anyone ever told {you/SP} that {you/she/he/SP} had yellow skin, yellow eyes or jaundice? Please do not include infant jaundice, which is common during the first weeks after birth.
40. How old {were you/was SP} when {you were/s/he was} first told {you/s/he} had yellow skin, yellow eyes or jaundice?
41. {Have you/Has SP} ever been told by a doctor or other health professional that {you/s/he} had cancer or a malignancy of any kind?
42. What kind of cancer was it?
43. How old (were you/was SP) when cancer was first diagnosed?
44. Including living and deceased, were any of {SP's/your} close biological that is, blood relatives including father, mother, sisters or brothers, ever told by a health professional that they had a heart attack or angina (an-gi-na) before the age of 50?
45. Including living and deceased, were any of {SP's/your} close biological that is, blood relatives including father, mother, sisters or brothers, ever told by a health professional that they had asthma (az-ma)?
46. Including living and deceased, were any of {SP's/your} close biological that is, blood relatives including father, mother, sisters or brothers, ever told by a health professional that they had diabetes?
47. To lower {your/SP's} risk for certain diseases, during the past 12 months {have you/has s/he} ever been told by a doctor or health professional to: control {your/his/her} weight or lose weight?
48. To lower {your/SP's} risk for certain diseases, during the past 12 months {have you/has s/he} ever been told by a doctor or health professional to: increase {your/his/her} physical activity or exercise?
49. To lower {your/SP's} risk for certain diseases, during the past 12 months {have you/has s/he} ever been told by a doctor or health professional to: reduce the amount of sodium or salt in {your/his/her} diet?
50. To lower {your/SP's} risk for certain diseases, during the past 12 months {have you/has s/he} ever been told by a doctor or health professional to: reduce the amount of fat or calories in {your/his/her} diet?
51. To lower {your/his/her} risk for certain diseases, {are you/is s/he} now doing any of the following: controlling {your/his/her} weight or losing weight?
52. To lower {your/his/her} risk for certain diseases, {are you/is s/he} now doing any of the following: increasing {your/his/her} physical activity or exercise?
53. To lower {your/his/her} risk for certain diseases, {are you/is s/he} now doing any of the following: reducing the amount of sodium or salt in {your/his/her} diet?

54. To lower {your/his/her} risk for certain diseases, {are you/is s/he} now doing any of the following: reducing the amount of fat or calories in {your/his/her} diet?
55. The following question is about metal objects you may have inside your body. Do you have any artificial joints, pins, plates, metal suture material, or other types of metal objects in your body? Some common examples are on the hand card.

5.21 Mental Health - Depression Screener

1. Over the last 2 weeks, how often have you been bothered by the following problems: little interest or pleasure in doing things?
2. [Over the last 2 weeks, how often have you been bothered by the following problems:] feeling down, depressed, or hopeless?
3. [Over the last 2 weeks, how often have you been bothered by the following problems:] trouble falling or staying asleep, or sleeping too much?
4. [Over the last 2 weeks, how often have you been bothered by the following problems:] feeling tired or having little energy?
5. [Over the last 2 weeks, how often have you been bothered by the following problems:] poor appetite or overeating?
6. [Over the last 2 weeks, how often have you been bothered by the following problems:] feeling bad about yourself - or that you are a failure or have let yourself or your family down?
7. [Over the last 2 weeks, how often have you been bothered by the following problems:] trouble concentrating on things, such as reading the newspaper or watching TV?
8. [Over the last 2 weeks, how often have you been bothered by the following problems:] moving or speaking so slowly that other people could have noticed? Or the opposite - being so fidgety or restless that you have been moving around a lot more than usual?
9. [Over the last 2 weeks, how often have you been bothered by the following problems:] Thoughts that you would be better off dead or of hurting yourself in some way?
10. How difficult have these problems made it for you to do your work, take care of things at home, or get along with people?

5.22 Occupation

1. (SP Interview Version) In this part of the survey I will ask you questions about {your/SP's} work experience. Which of the following {were you/was SP} doing last week . . . (Family Interview Version) The next questions are about {your/NON-SP HEAD'S/NON- SP SPOUSE'S} current job or business. Which of the following {were you/was} {NON-SP HEAD/NON-SP SPOUSE} doing last week . .
2. How many hours did {you/SP} work last week at all jobs or businesses?
3. {Do you/Does SP} usually work 35 hours or more per week in total at all jobs or businesses?
4. Looking at the card, which of these best describes this job or work situation?
5. About how long {have you/has SP} worked for {EMPLOYER} as a(n) {OCCUPATION}?

6. During the past 12 months at {your/SP's} job as a(n) {OCCUPATION} for {EMPLOYER}, how often {do you/does SP} wear protective hearing devices?
7. These next questions are about noise at work. First we are going to ask about loud noise. Loud means so loud that {you/s/he} must speak in a raised voice to be heard by someone three feet away when not using hearing protection. After that we will ask about very loud noise. Very loud noise is noise that is so loud {you have/he/she has} to shout to be heard by someone three feet away when not using hearing protection. How many days per month {are you/is SP} usually exposed to loud noise at {your/his/her} job as a(n) {OCCUPATION} for {EMPLOYER}? (Loud means so loud that {you/s/he} must speak in a raised voice to be heard by someone three feet away when not using hearing protection.)
8. On average, during the days when {you are/SP is} exposed to this loud noise, for how many hours per day {have you/has SP} been exposed? (Loud means so loud that {you/s/he} must speak in a raised voice to be heard by someone three feet away when not using hearing protection.)
9. How many days per month {are you/is SP} usually exposed to very loud noise at {your/his/her} job as a(n) {OCCUPATION} for {EMPLOYER}? (Very loud noise is noise that is so loud {you have/he/she has} to shout to be heard by someone three feet away when not using hearing protection.)
10. On average, during the days when {you are/SP is} exposed to this very loud noise, for how many hours per day {have you/has he/she} been exposed? (Very loud noise is noise that is so loud {you have/he/she has} to shout in order to be understood by someone standing 3 feet away from {you/him/her} when you aren't wearing hearing protection.)
11. (SP Interview Version) What is the main reason {you/SP} did not work last week? (Family Interview Version) What is the main reason {you/NON-SP HEAD/NON-SP SPOUSE} did not work last week?
12. Thinking of all the paid jobs or businesses {you/SP} ever had, what kind of work {were you/was s/he} doing the longest? (For example, electrical engineer, stock clerk, typist, farmer.)
13. About how long did {you/SP} work at that job or business?

5.23 Oral Health

1. The next questions are about {your/SP's} teeth and gums. About how long has it been since {you/SP} last visited a dentist? Include all types of dentists, such as, orthodontists, oral surgeons, and all other dental specialists, as well as dental hygienists.
2. What was the main reason {you/SP} last visited the dentist?
3. During the past 12 months was there a time when (you/SP) needed dental care but could not get it at that time?
4. What were the reasons that (you/SP) could not get the dental care (you/she/he) needed?

5. We would like you to think of the time when {SP} started brushing {his/her} teeth either with your help or alone. At what age did {SP} start brushing {his/her} teeth?
6. At what age did {SP} start using toothpaste?
7. Has {SP} ever received prescription fluoride drops or fluoride tablets?
8. How old in months or years was {SP} when {he/she} started taking prescription fluoride drops or fluoride tablets?
9. How old in months or years was {SP} when {he/she} stopped taking prescription fluoride drops or fluoride tablets?
10. In the past 12 months, did a dentist, hygienist or other dental professional have a direct conversation with {you/SP} about... ...the benefits of giving up cigarettes or other types of tobacco to improve {your/SP's} dental health?
11. (In the past 12 months, did a dentist, hygienist or other dental professional have a direct conversation with {you/SP} about...) ... the dental health benefits of checking {your/his/her} blood sugar?
12. (In the past 12 months, did a dentist, hygienist or other dental professional have a direct conversation with {you/SP} about...) ...the importance of examining {your/his/her} mouth for oral cancer?
13. How often during the last year (have you/ has SP) had painful aching anywhere in (your/his/her) mouth?
14. How often during the last year {have you/has SP} had difficulty doing {your/his/her} usual jobs or attending school because of problems with {your/his/her} teeth, mouth or dentures? Would you say . . .
15. How often during the last year {have you/has SP} been self-conscious or embarrassed because of {your/his/her} teeth, mouth or dentures?
16. The next questions will ask about the condition of {your/SP's} teeth and some factors related to gum health. Gum disease is a common problem with the mouth. People with gum disease might have swollen gums, receding gums, sore or infected gums or loose teeth. {Do you/Does SP} think {you/s/he} might have gum disease?
17. Overall, how would {you/SP} rate the health of {your/his/her} teeth and gums?
18. How many times {do you/does SP} brush (your/his/her} teeth in one day?
19. On average, how much toothpaste {do you/does SP} use when brushing {your/his/her} teeth?
20. {Have you/Has SP} ever had treatment for gum disease such as scaling and root planing, sometimes called "deep cleaning"
21. {Have you/Has SP} ever been told by a dental professional that {you/s/he} lost bone around [your/his/her} teeth?
22. Aside from brushing {your/his/her} teeth with a toothbrush, in the last seven days, how many days did {you/SP} use dental floss or any other device to clean between {your/his/her} teeth?
23. {Have you/Has SP} ever had an exam for oral cancer in which the doctor or dentist pulls on {your/his/her} tongue, sometimes with gauze wrapped around it, and feels under the tongue and inside the cheeks?

24. When did {you/SP} have {your/his/her} most recent oral or mouth cancer exam? Was it within the past year, between 1 and 3 years ago, or over 3 years ago?
25. What type of health care professional performed {your/SP's} most recent oral cancer exam?

5.24 Physical Activity

1. Next I am going to ask you about the time {you spend/SP spends} doing different types of physical activity in a typical week. Think first about the time {you spend/he spends/she spends} doing work. Think of work as the things that {you/he/she has} to do such as paid or unpaid work, household chores, and yard work. Does {your/SP's} work involve vigorous-intensity activity that causes large increases in breathing or heart rate like carrying or lifting heavy loads, digging or construction work for at least 10 minutes continuously?
2. In a typical week, on how many days {do you/does SP} do vigorous-intensity activities as part of {your/his/her} work?
3. How much time {do you/does SP} spend doing vigorous-intensity activities at work on a typical day?
4. Does {your/SP's} work involve moderate-intensity activity that causes small increases in breathing or heart rate such as brisk walking or carrying light loads for at least 10 minutes continuously?
5. In a typical week, on how many days {do you/does SP} do moderate-intensity activities as part of {your/his/her} work?
6. How much time {do you/does SP} spend doing moderate-intensity activities at work on a typical day?
7. The next questions exclude the physical activity at work that you have already mentioned. Now I would like to ask you about the usual way {you travel/SP travels} to and from places. For example, to school, for shopping, to work. In a typical week {do you/does SP} walk or use a bicycle for at least 10 minutes continuously to get to and from places?
8. In a typical week, on how many days {do you/does SP} walk or bicycle for at least 10 minutes continuously to get to and from places?
9. How much time {do you/does SP} spend walking or bicycling for travel on a typical day?
10. The next questions exclude the work and transport activities that you have already mentioned. Now I would like to ask you about sports, fitness and recreational activities. In a typical week {do you/does SP} do any vigorous-intensity sports, fitness, or recreational activities that cause large increases in breathing or heart rate like running or basketball for at least 10 minutes continuously?
11. In a typical week, on how many days {do you/does SP} do vigorous-intensity sports, fitness or recreational activities?
12. How much time {do you/does SP} spend doing vigorous-intensity sports, fitness or recreational activities on a typical day?

13. In a typical week {do you/does SP} do any moderate-intensity sports, fitness, or recreational activities that cause a small increase in breathing or heart rate such as brisk walking, bicycling, swimming, or volleyball for at least 10 minutes continuously?
14. In a typical week, on how many days {do you/does SP} do moderate-intensity sports, fitness or recreational activities?
15. How much time {do you/does SP} spend doing moderate-intensity sports, fitness or recreational activities on a typical day?
16. The following question is about sitting at school, at home, getting to and from places, or with friends including time spent sitting at a desk, traveling in a car or bus, reading, playing cards, watching television, or using a computer. Do not include time spent sleeping. How much time {do you/does SP} usually spend sitting on a typical day?
17. I'd like to ask you some questions about {your/SP's} activities. During the past 7 days, on how many days {were you/was SP} physically active for a total of at least 60 minutes per day? Add up all the time {you/he/she} spent in any kind of physical activity that increased {your/his/her} heart rate and made {you/him/her} breathe hard some of the time.
18. Now I will ask you first about TV watching and then about computer use. Over the past 30 days, on average how many hours per day did {you/SP} sit and watch TV or videos? Would you say . . .
19. Over the past 30 days, on average how many hours per day did {you/SP} use a computer or play computer games outside of school? Include Playstation, Nintendo DS, or other portable video games Would you say . . .
20. For the next questions, think about the sports, lessons, or physical activities {you/SP} may have done during the past 7 days? Please do not include things {you/he/she} did during the school day like PE or gym class. Did {you/SP} do any physical activities during the past 7 days?
21. What physical activities did {you/SP} do during the past 7 days? {PROBE: Did {you/he/she} do any other physical activities?}
22. During the past 7 days, on how many days did {you/SP} play active video games such as Wii Sports, Wii Fit, Xbox 360, Xbox Kinect, Playstation 3, or Dance, Dance Revolution?
23. On average, for how long did {you/SP} play these active video games?
24. In this question you can include activities done in school. On how many of the past 7 days did {you/SP} exercise or participate in physical activity for at least 20 minutes that made {you/him/her} sweat and breathe hard, such as basketball, soccer, running, swimming laps, fast bicycling, fast dancing, or similar activities?
25. On how many of the past 7 days did {you/SP} do exercises to strengthen or tone {your/his/her} muscles, such as push-ups, sit-ups, or weight lifting?
26. The next questions ask about activities during the school year. If {you are/SP is} not currently in school, think about {your/his/her} activities when {you/he/she was} last in school. Are students at {your/his/her} school allowed to use school facilities during lunch or during a free or elective period, such as the gymnasium, tennis courts, weight room, or track, during school time?
27. {Do you/Does SP} use school facilities for physical activity during school time?

28. {Do you/does SP} have PE or gym during school days?
29. How often {do you/does SP} have PE or gym?
30. On average, how long is the PE or gym class?
31. The following are activities that may be done before, during, or after school other than during PE or gym class. If {you are/SP is} not currently in school, think about {your/his/her} activities when {you/he/she was} last in school.} {Do you/Does SP} participate in school sports or physical activity clubs?
32. In what school sports or physical activity clubs {do you/does SP} participate?
33. Participate in martial arts
34. {Do you/Does SP} have recess during school days?
35. How often {do you/does SP} have recess?
36. On average, how long is the recess period?
37. About how many minutes {do you/does SP} think you should exercise or be physically active each day for good health?
38. I am going to read a statement and I want you to let me know if you strongly agree, agree, neither agree nor disagree, disagree or strongly disagree with the statement. {I enjoy participating in PE or gym class.}
39. In the past year, did {you/SP} receive a Physical Fitness Test award, such as a President's Challenge or Fitnessgram award?
40. What Physical Fitness Test award did {you/SP} receive?

5.25 Physical Functioning

1. {Do you/Does SP} have an impairment or health problem that limits {your/his/her} ability to {walk, run or play} {walk or run}?
2. Is this impairment or health problem that has lasted, or is expected to last 12 months or longer?
3. Both males and females 3 YEARS - 19 YEARS
4. {Do you/Does SP} have any impairment or health problem that requires {you/him/her} to use special equipment, such as a brace, a wheelchair, or a hearing aid (excluding ordinary eyeglasses or corrective shoes)?
5. Both males and females 3 YEARS - 19 YEARS
6. Does {SP} receive Special Education or Early Intervention Services?
7. The next set of questions is about limitations caused by any long-term physical, mental or emotional problem or illness. Please do not include temporary conditions, such as a cold [or pregnancy]. Does a physical, mental or emotional problem now keep {you/SP} from working at a job or business?
8. {Are you/Is SP} limited in the kind or amount of work {you/s/he} can do because of a physical, mental or emotional problem?
9. Because of a health problem, {do you/does SP} have difficulty walking without using any special equipment?
10. {Are you/Is SP} limited in any way because of difficulty remembering or because {you/s/he} experience{s} periods of confusion?

11. Both males and females 20 YEARS - 150 YEARS
12. {Are you/Is SP} limited in any way in any activities because of a physical, mental or emotional problem?
13. Both males and females 20 YEARS - 150 YEARS
14. The next questions ask about difficulties {you/SP} may have doing certain activities because of a health problem. By "health problem" we mean any long-term physical, mental or emotional problem or illness {not including pregnancy}. By {yourself/himself /herself} and without using any special equipment, how much difficulty {do you/does SP} have . . .managing {your/his/her} money [such as keeping track of {your/his/her} expenses or paying bills]?
15. By {yourself/himself/herself} and without using any special equipment, how much difficulty {do you/does SP} have . . .walking for a quarter of a mile [that is about 2 or 3 blocks]?
16. By {yourself/himself/herself} and without using any special equipment, how much difficulty {do you/does SP} have . . .walking up 10 steps without resting?
17. By {yourself/himself/herself} and without using any special equipment, how much difficulty {do you/does SP} have . . .stooping, crouching, or kneeling?
18. By {yourself/himself/herself} and without using any special equipment, how much difficulty {do you/does SP} have . . .lifting or carrying something as heavy as 10 pounds [like a sack of potatoes or rice]?
19. By {yourself/himself/herself} and without using any special equipment, how much difficulty {do you/does SP} have . . .doing chores around the house [like vacuuming, sweeping, dusting, or straightening up]?
20. By {yourself/himself/herself} and without using any special equipment, how much difficulty {do you/does SP} have . . .preparing {your/his/her} own meals?
21. By {yourself/himself/herself} and without using any special equipment, how much difficulty {do you/does SP} have . . .walking from one room to another on the same level?
22. By {yourself/himself/herself} and without using any special equipment, how much difficulty {do you/does SP} have . . .standing up from an armless straight chair?
23. By {yourself/himself/herself} and without using any special equipment, how much difficulty {do you/does SP} have . . .getting in or out of bed?
24. By {yourself/himself/herself} and without using any special equipment, how much difficulty {do you/does SP} have . . .eating, like holding a fork, cutting food or drinking from a glass?
25. By {yourself/himself/herself} and without using any special equipment, how much difficulty {do you/does SP} have . . .dressing {yourself/himself/herself}, including tying shoes, working zippers, and doing buttons?
26. By {yourself/himself/herself} and without using any special equipment, how much difficulty {do you/does SP} have . . .standing or being on {your/his/her} feet for about 2 hours?
27. By {yourself/himself/herself} and without using any special equipment, how much difficulty {do you/does SP} have . . .sitting for about 2 hours?

28. By {yourself/himself/herself} and without using any special equipment, how much difficulty {do you/does SP} have . . .reaching up over {your/his/her} head?
29. By {yourself/himself/herself} and without using any special equipment, how much difficulty {do you/does SP} have . . .using {your/his/her} fingers to grasp or handle small objects?
30. By {yourself/himself/herself} and without using any special equipment, how much difficulty {do you/does SP} have . . .going out to things like shopping, movies, or sporting events?
31. By {yourself/himself/herself} and without using any special equipment, how much difficulty {do you/does SP} have . . .participating in social activities [visiting friends, attending clubs or meetings or going to parties]?
32. By {yourself/himself/herself} and without using any special equipment, how much difficulty {do you/does SP} have . . .doing things to relax at home or for leisure [reading, watching TV, sewing, listening to music]?
33. By {yourself/himself/herself} and without using any special equipment, how much difficulty {do you/does SP} have . . .pushing or pulling large objects like a living room chair?
34. Both males and females 20 YEARS - 150 YEARS
35. What condition or health problem causes {you/SP} to have difficulty with or need help with {NAME OF UP TO 3 ACTIVITIES/these activities}?
36. {Do you/Does SP} now have any health problem that requires {you/him/her} to use special equipment, such as a cane, a wheelchair, a special bed, or a special telephone?

5.26 Prescription Medications

1. In the past 30 days, have you used or taken medication for which a prescription is needed? Do not include prescription vitamins or minerals you may have already told me about.
2. Generic drug name.
3. Generic drug code.
4. Was prescription container seen by interviewer?
5. For how long have you been using or taking {PRODUCT NAME}?
6. ICD-10-CM code 1.
7. ICD-10-CM code 2.
8. ICD-10-CM code 3.
9. ICD-10-CM code 1 description.
10. ICD-10-CM code 2 description.
11. ICD-10-CM code 3 description.
12. The number of prescription medicines reported.

5.27 Preventive Aspirin Use

1. Doctors and other health care providers sometimes recommend that {you take/SP takes) a low-dose aspirin each day to prevent heart attacks, strokes, or cancer. {Have you/Has SP} ever been told to do this?
2. {Are you/Is SP} now following this advice?
3. On {your/SP's} own, {are you/is SP} now taking a low-dose aspirin each day to prevent heart attacks, strokes, or cancer?
4. How often {do you/does SP} take an aspirin? (ASA taken daily, on alternate days, or another schedule?)
5. How often {do you/does SP} take an aspirin? (Number of ASA doses taken per day or per week).
6. How often {do you/does SP} take an aspirin? (ASA doses taken on a daily or weekly basis?)
7. What is the size or dose that {you take/SP takes}?

5.28 Reproductive Health

1. The next series of questions are about {your/SP's} reproductive history. I will begin by asking about {your/SP's} periods or menstrual cycles. How old {were you/was SP} when {you/SP} had {your/her} first menstrual period?
2. {Were you/Was SP}...
3. {Have you/Has SP} had at least one menstrual period in the past 12 months? (Please do not include bleedings caused by medical conditions, hormone therapy, or surgeries.)
4. What is the reason that {you have/SP has} not had a period in the past 12 months?
5. About how old {were you/was SP} when {you/SP} had {your/her} last menstrual period?
6. The next questions are about {your/SP's} pregnancy history. {Have you/Has SP} ever attempted to become pregnant over a period of at least a year without becoming pregnant?
7. {Have you/Has SP} ever been to a doctor or other medical provider because {you have/she has} been unable to become pregnant?
8. {Have you/Has SP} ever been treated for an infection in {your/her} fallopian tubes, uterus or ovaries, also called a pelvic infection, pelvic inflammatory disease, or PID?
9. The next questions are about {your/SP's} pregnancy history. {Have you/Has SP ever been pregnant? Please include (current pregnancy,) live births, miscarriages, stillbirths, tubal pregnancies and abortions.
10. {Are you/Is SP} pregnant now?
11. How many times {have you/has SP} been pregnant? ({Again, be/Be} sure to count all {your/her} pregnancies including (current pregnancy,) live births, miscarriages, stillbirths, tubal pregnancies or abortions.)
12. During {any/your/SP's} pregnancy, {were you/was SP} ever told by a doctor or other health professional that {you/she} had diabetes, sugar diabetes or gestational diabetes?

Please do not include diabetes that {you/SP} may have known about before the pregnancy.

13. How old {were you/was SP} when {you were/she was} first told {you/she} had diabetes during a pregnancy?
14. How many vaginal deliveries {have you/has SP} had? {Please count stillbirths as well as live births}
15. How many cesarean deliveries {have you/has SP} had? (Cesarean deliveries are also known as C-sections.) (Please count stillbirths as well as live births.)
16. {Did {your/SP's} delivery/Did any of {your/SP's} deliveries} result in a baby that weighed 9 pounds (4082 g) or more at birth? (Please count stillbirths as well as live births.)
17. How old {were you/was SP} when {you/she} delivered a baby that weighs 9 pounds or more? (Please count stillbirths as well as live births.)
18. How many of {your/her} deliveries resulted {Did {your/her} delivery result} in a live birth?
19. How old {were you/was SP} at the time of {your/her} first live birth?
20. How old {were you/was SP} at the time of {your/her} last live birth?
21. How many months ago did {you/SP} have {your/her} baby?
22. {Are you/Is SP} now breast feeding a child?
23. {Have you/Has SP} had a hysterectomy that is, surgery to remove {your/her} uterus or womb?
24. How old {were you/was SP} when {you/she} had {your/her} (hysterectomy/uterus removed/womb removed)?
25. {Have you/Has SP} had both of {your/her} ovaries removed (either when {you/she} had {your/her} uterus removed or at another time)?
26. How old {were you/was SP} when {you/she} had {your/her} ovaries removed or last ovary removed if removed at different times?
27. Now I am going to ask you about {your/SP's} birth control history. {Have you/Has SP} ever taken birth control pills for any reason?
28. {Have you/Has SP} ever used female hormones such as estrogen and progesterone? Please include any forms of female hormones, such as pills, cream, patch, and injectables, but do not include birth control methods or use for infertility.
29. Which forms of female hormones {have you/has SP} used.
30. {Have you/Has SP} ever taken female hormone pills containing estrogen only (like Premarin)? (Do not include birth control pills.)
31. Not counting any time when {you/SP} stopped taking them, for how long altogether {have you taken/did you take/has she taken/did she take} pills containing estrogen only?
32. {Have you/Has SP} taken female hormone pills containing both estrogen and progestin (like Prempro, Premphase)? (Do not include birth control pills.)
33. Not counting any time when {you/SP} stopped taking them, for how long altogether {{have you/has SP} taken/did {you/SP} take} pills containing both estrogen and progestin?
34. {Have you/Has SP} ever used female hormone patches containing estrogen only?

35. Not counting any time when {you/SP} stopped taking them, for how long altogether {have you used/did you use/has she used/did she use} patches containing estrogen only?
36. {Have you/Has SP} used female hormone patches containing both estrogen and progestin?
37. Not counting any time when {you/SP} stopped taking them, for how long altogether {have you used/did you use/has she used/did she use} patches containing both estrogen and progestin?

5.29 Sexual Behavior

1. Ever had vaginal, anal, or oral sex.
2. Have you ever had vaginal sex, also called sexual intercourse, with a woman? This means your penis in a woman's vagina.
3. Have you ever performed oral sex on a woman? This means putting your mouth on a woman's vagina or genitals.
4. Have you ever had anal sex with a woman? Anal sex means contact between your penis and a woman's anus or butt.
5. Have you ever had any kind of sex with a man, including oral or anal?
6. Have you ever had vaginal sex, also called sexual intercourse, with a man? This means a man's penis in your vagina.
7. Have you ever performed oral sex on a man? This means putting your mouth on a man's penis or genitals.
8. Have you ever had anal sex? This means contact between a man's penis and your anus or butt.
9. Have you ever had any kind of sex with a woman? By sex, we mean sexual contact with another woman's vagina or genitals.
10. How old were you when you had sex for the first time?
11. In your lifetime, with how many women have you had any kind of sex?
12. In the past 12 months, with how many women have you had any kind of sex?
13. In your lifetime, with how many women have you had vaginal sex? Vaginal sex means your penis in a woman's vagina.
14. In the past 12 months, with how many women have you had vaginal sex? Vaginal sex means your penis in a woman's vagina.
15. How old were you when you first performed oral sex on a woman performing oral sex means your mouth on a woman's vagina or genitals.
16. In your lifetime, on how many women have you performed oral sex?
17. In the past 12 months, on how many women have you performed oral sex?
18. How long has it been since the last time you performed oral sex on a new female partner? A new sexual partner is someone that you had never had sex with before.
19. In your lifetime, with how many men have you had anal or oral sex?
20. In the past 12 months, with how many men have you had anal or oral sex?
21. In your lifetime, with how many men have you had anal sex?

22. In the past 12 months, with how many men have you had anal sex?
23. Have you ever performed oral sex on a man? Performing oral sex means your mouth on a man's penis or genitals.
24. How old were you when you first performed oral sex on a man? Performing oral sex means your mouth on a man's penis or genitals.
25. In your lifetime, on how many men have you performed oral sex?
26. In the past 12 months, on how many men have you performed oral sex?
27. How long has it been since the last time you performed oral sex on a new male partner? A new sexual partner is someone that you had never had sex with before.
28. When you performed oral sex in the past 12 months, how often would you use protection, like a condom or dental dam?
29. In the past 12 months, did you have any kind of sex with a person that you never had sex with before?
30. In the past 12 months, about how many times have you had {vaginal or anal/vaginal/anal} sex?
31. In the past 12 months, about how often have you had {vaginal or anal/vaginal/anal} sex without using a condom?
32. Of the persons you had any kind of sex with in the past 12 months, how many were five or more years older than you?
33. Of the persons you had any kind of sex with in the past 12 months, how many were five or more years younger than you?
34. In your lifetime, with how many men have you had any kind of sex?
35. In the past 12 months, with how many men have you had any kind of sex?
36. In your lifetime, with how many men have you had vaginal sex? Vaginal sex means a man's penis in your vagina.
37. In the past 12 months, with how many men have you had vaginal sex? Vaginal sex means a man's penis in your vagina.
38. In your lifetime with how many women have you had sex? By sex, we mean sexual contact with another woman's vagina or genitals.
39. In the past 12 months, with how many women have you had sex? By sex, we mean sexual contact with another woman's vagina or genitals.
40. Have you ever performed oral sex on a woman? Performing oral sex means your mouth on a woman's vagina or genitals.
41. Has a doctor or other health care professional ever told you that you had human papillomavirus or HPV?
42. Has a doctor or other health care professional ever told you that you had genital herpes?
43. Has a doctor or other health care professional ever told you that you had genital warts?
44. How old were you when you were first told that you had genital warts?
45. In the past 12 months, has a doctor or other health care professional told you that you had gonorrhea, sometimes called GC or clap?
46. In the past 12 months, has a doctor or other health care professional told you that you had chlamydia?
47. Are you circumcised or uncircumcised?

48. Do you think of yourself as...
49. Which of the following best represents how you think of yourself?

5.30 Sleep Disorders

1. The next set of questions is about {your/SP's} sleeping habits. The first two questions refer to the times {you get/SP gets} in and out of bed in order to sleep, not including naps. What time {do you/does SP} usually go to sleep on weekdays or workdays?
2. What time {do you/does SP} usually wake up on weekdays or workdays?
3. How much sleep {do you/does SP} usually get at night on weekdays or workdays?
4. In the past 12 months, how often did {you/SP} snore while {you were/s/he was} sleeping?
5. In the past 12 months, how often did {you/SP} snort, gasp, or stop breathing while {you were/s/he was} asleep?
6. {Have you/Has SP} ever told a doctor or other health professional that {you have/s/he has} trouble sleeping?
7. In the past month, how often did {you/SP} feel excessively or overly sleepy during the day?

5.31 Smoking - Cigarette Use

1. These next questions are about cigarette smoking and other tobacco use. {Have you/Has SP} smoked at least 100 cigarettes in {your/his/her} entire life?
2. How old {were you/was SP} when {you/s/he} first started to smoke cigarettes fairly regularly?
3. {Do you/Does SP} now smoke cigarettes?
4. How long has it been since {you/SP} quit smoking cigarettes?
5. UNIT OF MEASURE
6. How old {were you/was SP} when {you/s/he} last smoked cigarettes {fairly regularly}?
7. At that time, about how many cigarettes did {you/SP} usually smoke per day?
8. How soon after you wake up do you smoke? Would you say . . .
9. On how many of the past 30 days did {you/SP} smoke a cigarette?
10. During the past 30 days, on the days that {you/SP} smoked, about how many cigarettes did {you/s/he} smoke per day?
11. May I please see the pack for the brand of cigarettes {you usually smoke/SP usually smokes}.
12. Cigarette 12-digit Universal Product Code (UPC)
13. BRAND OF CIGARETTES SMOKED BY SP (SUB-BRAND INCLUDED IF APPLICABLE AND AVAILABLE)
14. CIGARETTE PRODUCT FILTERED OR NON-FILTERED
15. CIGARETTE PRODUCT MENTHOLATED OR NON-MENTHOLATED
16. CIGARETTE PRODUCT LENGTH
17. CIGARETTE TAR CONTENT

18. CIGARETTE NICOTINE CONTENT
19. CIGARETTE CARBON MONOXIDE CONTENT
20. The following questions are about cigarette smoking and other tobacco use. Do not include cigars or marijuana. About how many cigarettes have you smoked in your entire life?
21. How old were you when you smoked a whole cigarette for the first time?
22. During the past 30 days, on the days that you smoked, which brand of cigarettes do you usually smoke?
23. Please select the Marlboro pack that looks most like the brand that you smoke. If the pack you smoke is not shown, select 'other Marlboro.'
24. Please select the Camel pack that looks most like the brand that you smoke. If the pack you smoke is not shown, select 'other Camel.'
25. Please select the Newport pack that looks most like the brand that you smoke. If the pack you smoke is not shown, select 'other Newport.'
26. Please select the pack that looks most like the brand that you smoke. If the pack you smoke is not shown, select 'other brands of cigarettes.'
27. During the past 12 months, have you stopped smoking for one day or longer because you were trying to quit smoking?
28. During the past 12 months, how many times {have you/has SP} stopped smoking cigarettes because {you/he/she was} trying to quit smoking?
29. The last time {you/SP} tried to quit, how long {were you/he/she} able to stop smoking?
30. The last time {you/SP} tried to quit, how long {were you/he/she} able to stop smoking?
31. {Have you/Has SP} ever smoked a cigarette even one time?
32. How old {were you/was SP} the first time {you/he/she} smoked all or part of a cigarette?
33. {Do you/Does SP} now smoke cigarettes?
34. On how many of the past 30 days did {you/SP} smoke a cigarette?
35. {Have you/Has SP} ever smoked a regular cigar, cigarillo or little filtered cigar even one time? This hand card shows examples of some cigars; however there are others not included here.
36. During the past 30 days, on how many days did {you/SP} smoke a regular cigar, cigarillo or little filtered cigar?
37. The next question is about e-cigarettes. These are battery-powered devices that usually contains liquid nicotine, and don't produce smoke. Have {you/SP} EVER used an e-cigarette EVEN ONE TIME? This hand card shows examples of some e-cigarettes and other devices used to inhale liquid nicotine; however there are others not included here.
38. During the past 30 days, on how many days did {you/SP} use e-cigarettes?
39. Smokeless tobacco products are placed in the mouth and nose and include chewing tobacco, snuff, dip, snus (pronounced as "snoose") and dissolvable tobacco. {Have you/Has SP} ever used smokeless tobacco even one time? This hand card shows examples of smokeless products; however there are others not included here.
40. During the past 30 days, on how many days did {you/SP} use smokeless tobacco?

5.32 Smoking - Household Smokers

1. Now I would like to ask you a few questions about smoking in this home. How many people who live here smoke cigarettes, cigars, little cigars, pipes, water pipes, hookah, or any other tobacco product?
2. Not counting decks, porches, or detached garages, how many people who live here smoke cigarettes, cigars, little cigars, pipes, water pipes, hookah, or any other tobacco product inside this home?
3. (Not counting decks, porches, or detached garages) During the past days, that is since last [TODAY'S DAY OF WEEK], on how many days did {anyone who lives here/you}, smoke tobacco inside this home?

5.33 Smoking - Recent Tobacco Use

1. The following questions ask about the use of tobacco products in the past 5 days. During the past 5 days, including today, did you smoke cigarettes, pipes, cigars, little cigars or cigarillos, water pipes, hookahs, or e-cigarettes?
2. Which of these products did {you/he/she} use?
3. During the past 5 days, including today, on how many days did {you/he/she} smoke cigarettes?
4. During the past 5 days, including today, on the days {you/he/she} smoked, how many cigarettes did {you/he/she} smoke each day?
5. When did {you/he/she} smoke {your/his/her} last cigarette? Was it...
6. Which of these products did {you/he/she} use?
7. During the past 5 days, including today, on how many days did {you/he/she} smoke a pipe?
8. Which of these products did {you/he/she} use?
9. During the past 5 days, including today, on how many days did {you/he/she} smoke cigars, or little cigars or cigarillos?
10. Which of these products did {you/he/she} use?
11. During the past 5 days, including today, on how many days did {you/he/she} smoke tobacco in a water pipe or Hookah?
12. Which of these products did {you/he/she} use?
13. During the past 5 days, including today, on how many days did {you/he/she} smoke an e-cigarette?
14. Smokeless tobacco products are placed in the mouth or nose and include chewing tobacco, snuff, snus, or dissolvables. During the past 5 days, including today, did {you/he/she} use any smokeless tobacco? (Please do not include nicotine replacement products like patches, gum, lozenge, or spray which are considered products to help {you/him/her} stop smoking.)
15. Which of these products did {you/he/she} use?

16. During the past 5 days, including today, on how many days did {you/he/she} use chewing tobacco, such as Redman, Levi Garrett or Beechnut?
17. Which of these products did {you/he/she} use?
18. During the past 5 days, including today, on how many days did {you/he/she} use snuff, such as Skoal, Skoal Bandits, or Copenhagen?
19. Which of these products did {you/he/she} use?
20. During the past 5 days, including today, on how many days did {you/he/she} use snus?
21. Which of these products did {you/he/she} use?
22. During the past 5 days, including today, on how many days did {you/he/she} use dissolvables such as strips or orbs?
23. During the past 5 days, including today, did {you/he/she} use any nicotine replacement therapy products such as nicotine patches, gum, lozenges, inhalers, or nasal sprays?
24. Which of these products did {you/he/she} use?
25. During the past 5 days, including today, on how many days did {you/he/she} use any nicotine replacement therapy products such as nicotine patches, gum, lozenges, inhalers, or nasal sprays?
26. When did {you/he/she} last use a nicotine replacement therapy product? Was it . . .
27. Used any tobacco product last 5 days?

5.34 Secondhand Smoke Exposure

1. I will now ask you about tobacco smoke in other places. During the last 7 days, {were you/was SP} working at a job or business outside of the home?
2. While {you were/SP was} working at a job or business outside of the home, did someone else smoke cigarettes or other tobacco products indoors?
3. {I will now ask you about smoking in other places.} During the last 7 days, did {you/SP} spend time in a restaurant?
4. While {you were/SP was} in a restaurant, did someone else smoke cigarettes or other tobacco products indoors?
5. During the last 7 days, {did you/SP} spend time in a bar?
6. While {you were/SP was} in a bar, did someone else smoke cigarettes or other tobacco products indoors?
7. During the last 7 days, did {you/SP} ride in a car or motor vehicle?
8. While {you were/SP was} riding in a car or motor vehicle, did someone else smoke cigarettes or other tobacco products?
9. During the last 7 days, did {you/SP} spend time in a home other than {your/his/her} own?
10. While {you were/SP was} in a home other than {your/his/her} own, did someone else smoke cigarettes or other tobacco products indoors?
11. During the last 7 days,{were you/was SP} in any other indoor area?
12. While {you were/SP was} in the other indoor area, did someone else smoke cigarettes or other tobacco products?

5.35 Volatile Toxicant

1. First, I would like to ask you a few questions about {your/SP's} home. Does {your/her/his} home has an attached garage?
2. Is the source of water for {your/her/his} home from a private well?
3. {Do you/Does she/Does he} currently store paints or fuels inside {your/her/his} home? Include {your/her/his} basement {and attached garage}.
4. {Do you/Does she/Does he} currently use moth balls, moth crystals or toilet bowl deodorizers inside {your/her/his} home?
5. In the last three days, did {you/she/he} inhale smoke from any source for 10 or more minutes?
6. When did {you/she/he} last spend 10 or more minutes inhaling smoke?
7. Now I am going to ask you a few questions about {your/SP's} activities over the last 48 hours. This means today or yesterday. In the last 48 hours, did {you/she/he} cook or bake with natural gas?
8. Now I am going to ask you a few questions about {your/SP's} activities over the last 48 hours. This means today or yesterday. How long ago, in hours, did {you/she/he} cook or bake with natural gas?
9. In the last 48 hours, did {you/she/he} pump gas into a car or other motor vehicle {yourself/herself/ himself}?
10. How long ago, in hours, did {you/she/he} pump gas into a car or other motor vehicle {yourself/herself/ himself}?
11. In the last 48 hours, did {you/she/he} spend any time at a swimming pool, in a hot tub, or in a steam room?
12. How long ago, in hours, has it been since {you/she/he} spent time at a swimming pool, in a hot tub, or in a steam room?
13. In the last 48 hours, did {you/she/he} use dry cleaning solvents, visit a dry cleaning shop or wear clothes that had been dry-cleaned within the last week?
14. How long ago, in hours, has it been since {you/she/he} used dry cleaning solvents, visited a dry cleaning shop or wore clothes that had been dry-cleaned within the last week?
15. In the last 48 hours, did {you/she/he} take a hot shower or bath for five minutes or longer?
16. How long ago, in hours, has it been since {your/SP's} last shower or hot bath?
17. In the last 48 hours, did {you/she/he} breathe fumes from freshly painted indoor surfaces, paints, paint thinner, or varnish?
18. How long ago, in hours, has it been since {you/she/he} breathed fumes from freshly painted indoor surfaces, paints, paint thinner, or varnish?
19. In the last 48 hours, did {you/she/he} breathe fumes from diesel fuel or kerosene?
20. How long ago, in hours, has it been since {you/she/he} breathed fumes from diesel fuel or kerosene?
21. In the last 48 hours, did {you/she/he} breathe fumes from fingernail polish?

22. How long ago, in hours, has it been since {you/she/he} breathed fumes from fingernail polish?

5.36 Weight History

1. These next questions ask about {your/SP's} height and weight at different times in {your/his/her} life. How tall {are you/is SP} without shoes?
2. How much {do you/does SP} weigh without clothes or shoes?
3. {Do you/Does SP} consider {your/his/her}self now to be . . . [If {you are/she is} currently pregnant, what did {you/she} consider {your/her}self to be before {you were/she was} pregnant?]
4. Would {you/SP} like to weigh . . .
5. How much did {you/SP} weigh a year ago?
6. Was the change between {your/SP's} current weight and {your/his/her} weight a year ago intentional?
7. During the past 12 months, {have you/has SP} tried to lose weight?
8. How did {you/SP} try to lose weight?
9. How many times {have you/has SP} lost 10 pounds or more because {you/he/she was} trying to lose weight? Was it . . .
10. How much did {you/SP} weigh 10 years ago? [If you don't know {your/his/her} exact weight, please make your best guess.]
11. How much did {you/SP} weigh at age 25? [If you don't know {your/his/her} exact weight, please make your best guess.] If (you were/she was) pregnant, how much did (you/she) weigh before (your/her) pregnancy?
12. How tall {were you/was SP} at age 25? [If you don't know {your/his/her} exact height, please make your best guess.]
13. Up to the present time, what is the most {you have/SP has} ever weighed?
14. How old {were you/was SP} then? [If you don't know {your/his/her} exact age, please make your best guess.]
15. {Have you/Has SP} ever had weight loss surgery, also called bariatric surgery?
16. [How old {were you/was SP} when {you/she/he} had {the most recent} weight loss surgery?]

5.37 Weight History - Youth

1. Do you consider yourself now to be . . .
2. Which of the following are you trying to do about your weight:
3. In the past year, how often have you tried to lose weight? Would you say . .

5.38 Demographics Data

Demographic Variables and Sample Weights

1. Respondent sequence number.
2. Data release cycle
3. Interview and examination status of the participant.
4. Gender of the participant.
5. Age in years of the participant at the time of screening. Individuals 80 and over are topcoded at 80 years of age.
6. Age in months of the participant at the time of screening. Reported for persons aged 24 months or younger at the time of exam (or screening if not examined).
7. Recode of reported race and Hispanic origin information
8. Recode of reported race and Hispanic origin information, with Non-Hispanic Asian Category
9. Six month time period when the examination was performed - two categories: November 1 through April 30, May 1 through October 31.
10. Age in months of the participant at the time of examination. Reported for persons aged 19 years or younger at the time of examination.
11. {Have you/Has SP} ever served on active duty in the U.S. Armed Forces, military Reserves, or National Guard? (Active duty does not include training for the Reserves or National Guard, but does include activation, for service in the U.S. or in a foreign country, in support of military or humanitarian operations.)
12. Did {you/SP} ever serve in a foreign country during a time of armed conflict or on a humanitarian or peace-keeping mission? (This would include National Guard or reserve or active duty monitoring or conducting peacekeeping operations in Bosnia and Kosovo, in the Sinai between Egypt and Israel, or in response to the 2004 tsunami or Haiti in 2010.)
13. In what country {were you/was SP} born?
14. {Are you/Is SP} a citizen of the United States? [Information about citizenship is being collected by the U.S. Public Health Service to perform health related research. Providing this information is voluntary and is collected under the authority of the Public Health Service Act. There will be no effect on pending immigration or citizenship petitions.]
15. Length of time the participant has been in the US.
16. What is the highest grade or level of school {you have/SP has} completed or the highest degree {you have/s/he has} received?
17. What is the highest grade or level of school {you have/SP has} completed or the highest degree {you have/s/he has} received?
18. Marital status
19. Pregnancy status for females between 20 and 44 years of age at the time of MEC exam.
20. Language of the Sample Person Interview Instrument
21. Was a Proxy respondent used in conducting the Sample Person (SP) interview?
22. Was an interpreter used to conduct the Sample Person (SP) interview?
23. Language of the Family Interview Instrument
24. Was a Proxy respondent used in conducting the Family Interview?
25. Was an interpreter used to conduct the Family interview?
26. Language of the MEC CAPI Interview Instrument

27. Was a Proxy respondent used in conducting the MEC CAPI Interview?
28. Was an interpreter used to conduct the MEC CAPI interview?
29. Language of the MEC ACASI Interview Instrument
30. Total number of people in the Household
31. Total number of people in the Family
32. Number of children aged 5 years or younger in the household
33. Number of children aged 6-17 years old in the household
34. Number of adults aged 60 years or older in the household
35. HH reference person's gender
36. HH reference person's age in years
37. HH reference person's country of birth
38. HH reference person's education level
39. HH reference person's marital status
40. HH reference person's spouse's education level
41. Full sample 2 year interview weight.
42. Full sample 2 year MEC exam weight.
43. Masked variance unit pseudo-PSU variable for variance estimation
44. Masked variance unit pseudo-stratum variable for variance estimation
45. Total household income (reported as a range value in dollars)
46. Total family income (reported as a range value in dollars)
47. A ratio of family income to poverty guidelines.
