# Supplementary material for: Machine Learning for Risk Group Identification and User Data Collection in a Herpes Simplex Virus Patient Registry: Algorithm Development and Validation Study
Source: JMIRx Med. 2021 Jun 11;2(2):e25560. doi: 10.2196/25560 (PMC10414389; doi:10.2196/25560)
Supplement: Multimedia Appendix 2 [file xmed_v2i2e25560_app2.pdf]

1. 'RIAGENDR': Gender of the participant.,
2. 'RIDAGEYR': Age in years of the participant at the time of screening. Individuals 80 and over are topcoded at 80 years of age.',
3. ‘DMQADFC’: Did you ever serve in a foreign country during a time of armed conflict or on a humanitarian or peace-keeping mission? (This would include National Guard or reserve or active duty monitoring or conducting peace keeping operations in Bosnia and Kosovo, in the Sinai between Egypt and Israel, or in response to the 2004 tsunami or Haiti in 2010.)
4. 'DMQMILIZ': Have you ever served on active duty in the Armed Forces, military Reserves, or National Guard?,
5. 'DMDEDUC3': What is the highest grade or level of school you have completed or the highest degree you have received?,
6. 'DMDEDUC2': What is the highest grade or level of school you have completed or the highest degree you have received?,
7. 'DMDMARTL': Marital status,
8. 'DMDHHSIZ': Total number of people in the Household,
9. 'DMDFMSIZ': Total number of people in the Family,
10. 'DMDHHSZA': Number of children aged 5 years or younger in the household,
11. 'DMDHHSZB': Number of children aged 6-17 years old in the household,
12. ‘DMDHHSZE’: Number of adults aged 60 years or older in the household
13. ‘DMDHRGND’: HH reference person's gender
14. ‘DMDHRAGE’: HH reference person's age in years
15. ‘DMDHRBR4’: HH reference person's country of birth
16. ‘DMDHREDU’: HH reference person's education level
17. ‘DMDHRMAR’: HH reference person's marital status
18. ‘DMDHSEDU’ : HH reference person's spouse's education level
19. 'INDHHIN2': Total household income,
20. 'INDFMIN2': Total family income,
21. ‘INQ020’: The next questions are about your income.
22. ‘INDFMMPI’: Family monthly poverty level index, a ratio of monthly family income to the HHS poverty guidelines specific to family size.
23. ‘INDFMMPC’: Family monthly poverty level index categories.
24. ‘IND235’: Monthly family income (reported as a range value in dollars).
25. 'INQ320': How do you usually get to the store where you do most of your grocery shopping?
26. ‘PAQ605’: Next I am going to ask you about the time you spend doing different types of physical activity in a typical week. Think first about the time you spend doing work. Think of work as the things that you have to do such as paid or unpaid work, household chores, and yard work. Does your work involve vigorous-intensity activity that causes large increases in breathing or heart rate like carrying or lifting heavy loads, digging or construction work for at least 10 minutes continuously?
27. ‘PAQ635’: The next questions exclude the physical activity at work that you have already mentioned. Now I would like to ask you about the usual way you travel to and from places.

28. ‘PAQ724a’:Physical activity aerobics
29. ‘PAQ724b’: Physical activity baseball
30. ‘PAQ724c’: Physical activity basketball
31. ‘PAQ724d’: Physical activity bike riding
32. ‘PAQ724e’: Physical activity cheerleading
33. ‘PAQ724f’: Physical activity dance
34. ‘PAQ724g’: Physical activity field hockey
35. ‘PAQ724h’: Physical activity football
36. ‘PAQ724i’: Physical activity golf
37. ‘PAQ724j’: Physical activity gymnastics
38. ‘PAQ724k’: Physical activity hiking
39. ‘PAQ724l’: Physical activity ice hockey
40. ‘PAQ724m’: Physical activity ice skating
41. ‘PAQ724n’: Physical activity jumping rope
42. ‘PAQ724o’: Physical activity lacrosse
43. ‘PAQ724q’: Physical activity martial arts
44. ‘PAQ724r’: Physical activity playing games
45. ‘PAQ724s’: Physical activity roller blading
46. ‘PAQ724t’: Physical activity running
47. ‘PAQ724u’: Physical activity scooter riding
48. ‘PAQ724v’: Physical activity skateboarding
49. ‘PAQ724w’: Physical activity soccer
50. ‘PAQ724x’: Physical activity swimming
51. ‘PAQ724y’: Physical activity tennis
52. ‘PAQ724z’: Physical activity track & field
53. ‘PAQ724aa’: Physical activity volleyball
54. ‘PAQ724ab’: Physical activity walking
55. ‘PAQ724ac’: Physical activity wrestling
56. ‘PAQ724ad’: Physical activity frisbee
57. ‘PAQ724ae’: Physical activity backyard games
58. ‘PAQ724af’: Physical activity trampoline
59. ‘PAQ724cm’: Physical activity other
60. ‘PAQ740’: The next questions ask about activities during the school year. If you are not currently in school, think about your activities when you were last in school. Are students at your school allowed to use school facilities during lunch or during a free or elective period, such as the gymnasium, tennis courts, weight room, or track, during school time?
61. ‘PAQ742’: Do you use school facilities for physical activity during school time?
62. ‘PAQ744’: Do you have PE or gym during school days?
63. ‘PAQ759S’: In what school sports or physical activity clubs do you participate?
64. 'PAQ650': In a typical week do you do any vigorous-intensity sports, fitness, or recreational activities that cause large increases in breathing or heart rate like running or basketball for at least 10 minutes continuously?,

65. 'PAQ665': In a typical week do you do any moderate-intensity sports, fitness, or recreational activities that cause a small increase in breathing or heart rate such as brisk walking, bicycling, swimming, or volleyball for at least 10 minutes continuously?,
66. 'PAQ620': Does your work involve moderate-intensity activity that causes small increases in breathing or heart rate such as brisk walking or carrying light loads for at least 10 minutes continuously?,
67. 'ALQ101': In any one year, have you had at least 12 drinks of any type of alcoholic beverage? By a drink, I mean a 12 oz. beer, a 5 oz. glass of wine, or a one and a half ounces of liquor.,
68. 'SXD021': Ever had vaginal, anal, or oral sex?,
69. ‘SXQ806’: Have you ever had anal sex with a woman? Anal sex means contact between your penis and a woman's anus or butt.
70. ‘SXQ703’: Have you ever performed oral sex on a man? This means putting your mouth on a man's penis or genitals.
71. ‘SXQ706’: Have you ever had anal sex? This means contact between a man's penis and your anus or butt.
72. ‘SXQ853’: Have you ever performed oral sex on a man? Performing oral sex means your mouth on a man's penis or genitals.
73. ‘SXQ260’: Has a doctor or other health care professional ever told you that you had genital herpes?
74. ‘SXQ265’:Has a doctor or other health care professional ever told you that you had genital warts?
75. ‘SXQ600’: Of the persons you had any kind of sex with in the past 12 months, how many were five or more years younger than you?
76. ‘SXQ130’: In your lifetime with how many women have you had sex? By sex, we mean sexual contact with another woman's vagina or genitals.
77. ‘SXQ490’: In the past 12 months, with how many women have you had sex? By sex, we mean sexual contact with another woman's vagina or genitals.
78. ‘SXQ741’: Have you ever performed oral sex on a woman? Performing oral sex means your mouth on a woman's vagina or genitals.
79. ‘SXQ841’: In the past 12 months, with how many men have you had anal sex?
80. ‘SXQ639’: In the past 12 months, on how many women have you performed oral sex?
81. 'SXQ800': Have you ever had vaginal sex, also called sexual intercourse, with a woman? This means your penis in a woman's vagina.,
82. 'SXQ803': Have you ever performed oral sex on a woman? This means putting your mouth on a woman's vagina or genitals.,
83. 'SXQ809': Have you ever had any kind of sex with a man, including oral or anal?,
84. 'SXQ700': Have you ever had vaginal sex, also called sexual intercourse, with a man? This means a man's penis in your vagina.,
85. 'SXQ550': In the past 12 months, with how many men have you had anal or oral sex?,
86. 'SXQ709': 'Have you ever had any kind of sex with a woman? By sex, we mean sexual contact with another woman's vagina or genitals.,
87. 'SXD031': How old when first had sex?
88. 'SXQ295': Describe sexual identity

89. 'SXQ836': In your lifetime, with how many men have you had anal sex?,
90. 'SXD171': 'In your lifetime, with how many women have you had any kind of sex?,
91. 'SXQ824': In your lifetime, with how many women have you had vaginal sex? Vaginal sex means your penis in a woman's vagina.,
92. 'SXD621': How old were you when you first performed oral sex on a man? Performing oral sex means your mouth on a man's penis or genitals.,
93. 'SXD630': How long has it been since the last time you performed oral sex on a new male partner? A new sexual partner is someone that you had never had sex with before.,
94. 'SXQ645': When you performed oral sex in the past 12 months, how often would you use protection, like a condom or dental dam?,
95. 'SXQ267': How old were you when you were first told that you had genital warts?,
96. 'SXQ627': In the past 12 months, on how many men have you performed oral sex?,
97. 'SXQ590': Of the persons you had any kind of sex with in the past 12 months, how many were five or more years older than you?,
98. 'SXD510': In the past 12 months, with how many women have you had any kind of sex?,
99. 'SXD633': How old were you when you first performed oral sex on a woman? Performing oral sex means your mouth on a woman's vagina or genitals.,
100. 'SXD101': In your lifetime, with how many men have you had any kind of sex?,
101. 'SXQ648': In the past 12 months, did you have any kind of sex with a person that you never had sex with before?,
102. 'SXQ624': In your lifetime, on how many women have you performed oral sex? (Male/Female),
103. 'SXQ610': In the past 12 months, about how many times have you had vaginal or anal sex?,
104. 'SXQ251': In the past 12 months, about how often have you had vaginal or anal sex without using a condom?,
105. 'SXD450': In the past 12 months, with how many men have you had any kind of sex?,
106. 'SXQ727': In the past 12 months, with how many men have you had vaginal sex? Vaginal sex means a man's penis in your vagina.,
107. 'SXQ753': Has a doctor or other health care professional ever told you that you had human papillomavirus or HPV?,
108. 'SXQ410': In your lifetime, with how many men have you had anal or oral sex?,
109. 'SXD642': How long has it been since the last time you performed oral sex on a new female partner? A new sexual partner is someone that you had never had sex with before.,
110. 'SXQ270': In the past 12 months, has a doctor or other health care professional told you that you had gonorrhea, sometimes called GC or clap?,
111. 'SXQ272': In the past 12 months, has a doctor or other health care professional told you that you had chlamydia?,
112. 'SXQ827': In the past 12 months, with how many women have you had vaginal sex? Vaginal sex means your penis in a woman's vagina.,
113. 'SXQ280': Circumcised or uncircumcised?,

114. 'SXQ636': In your lifetime, on how many women have you performed oral sex?,
115. 'SXQ724': In your lifetime, with how many men have you had vaginal sex? Vaginal sex means a man's penis in your vagina.,
116. 'SXQ296': Which of the following best represents how you think of yourself?
117. 'RXDUSE': In the past 30 days, have you used or taken medication for which a prescription is needed? Do not include prescription vitamins or minerals you may have already told me about.,
118. ‘RHQ020’: Age range at first menstrual period
119. ‘RHQ031’: Have you had at least one menstrual period in the past 12 months? (Please do not include bleedings caused by medical conditions, hormone therapy, or surgeries.)
120. ‘RHD043’: What is the reason that you have not had a period in the past 12 months?
121. 'RHQ074': The next questions are about your pregnancy history. Have you ever attempted to become pregnant over a period of at least a year without becoming pregnant?,
122. ‘RHQ200’: Are you now breast feeding a child?
123. ‘RHQ420’: Had both ovaries removed?
124. ‘RHQ542A’:Hormone pills used
125. ‘RHQ542B’: Hormone patches used
126. ‘RHQ542C’: Hormone cream/suppository/injection used
127. ‘RHQ542D’: Other form of female hormone used
128. ‘RHQ554’: Have you ever taken female hormone pills containing estrogen only (like Premarin)? (Do not include birth control pills.)
129. ‘RHQ570’: Have you taken female hormone pills containing both estrogen and progestin (like Prempro, Premphase)? (Do not include birth control pills.)
130. ‘RHQ580’: Have you ever used female hormone patches containing estrogen only?
131. ‘RHQ596’: Have you used female hormone patches containing both estrogen and progestin?
132. 'RHQ078': Have you ever been treated for an infection in your fallopian tubes, uterus or ovaries, also called a pelvic infection, pelvic inflammatory disease, or PID?,
133. 'RHQ131': The next questions are about your pregnancy history. Have you ever been pregnant? Please include (current pregnancy,) live births, miscarriages, stillbirths, tubal pregnancies and abortions.,
134. 'RHQ160': How many times have you been pregnant? (Again/Be sure to count all your pregnancies including (current pregnancy,) live births, miscarriages, stillbirths, tubal pregnancies or abortions.),
135. 'RHQ169': How many cesarean deliveries have you had? (Cesarean deliveries are also known as C-sections.) (Please count stillbirths as well as live births.)',
136. 'RHQ166': How many vaginal deliveries have you had? (Please count stillbirths as well as live births)',
137. 'RHQ171': How many of your deliveries resulted Did your delivery result in a live birth?,

138. 'RHD143': Are you pregnant now?,
139. 'RHD180': How old were you at the time of your first live birth?,
140. 'RHD190': How old were you at the time of your last live birth?,
141. 'RHQ540': Have you ever used female hormones such as estrogen and progesterone? Please include any forms of female hormones, such as pills, cream, patch, and injectables, but do not include birth control methods or use for infertility.,
142. 'RHQ305': Had both ovaries removed?,
143. 'SLD012': How much sleep do you usually get at night on weekdays or workdays?,
144. 'SMQ020': These next questions are about cigarette smoking and other tobacco use. Have you smoked at least 100 cigarettes in your entire life?,
145. 'SMD030': How old were you when you first started to smoke cigarettes fairly regularly?,
146. 'SMQ900': The next question is about e-cigarettes. These are battery-powered devices that usually contains liquid nicotine, and don't produce smoke. Have you EVER used an e-cigarette EVEN ONE TIME? This hand card shows examples of some e-cigarettes and other devices used to inhale liquid nicotine; however there are others not included here,
147. 'SMQ910': Smokeless tobacco products are placed in the mouth and nose and include chewing tobacco, snuff, dip, snus (pronounced as "snoose") and dissolvable tobacco. Have you ever used smokeless tobacco even one time? This hand card shows examples of smokeless products; however there are others not included here.,
148. 'SMQ890': Have you ever smoked a regular cigar, cigarillo or little filtered cigar even one time? This hand card shows examples of some cigars; however there are others not included here,
149. 'Flu':Is your general feeling of discomfort or illness followed by one or more symptoms: fever, nausea, headaches, muscle pain, swollen lymph nodes and malaise?
