# Supplementary figures and images for: Machine Learning for Risk Group Identification and User Data Collection in a Herpes Simplex Virus Patient Registry: Algorithm Development and Validation Study
Source: JMIRx Med. 2021 Jun 11;2(2):e25560. doi: 10.2196/25560 (PMC10414389; doi:10.2196/25560)

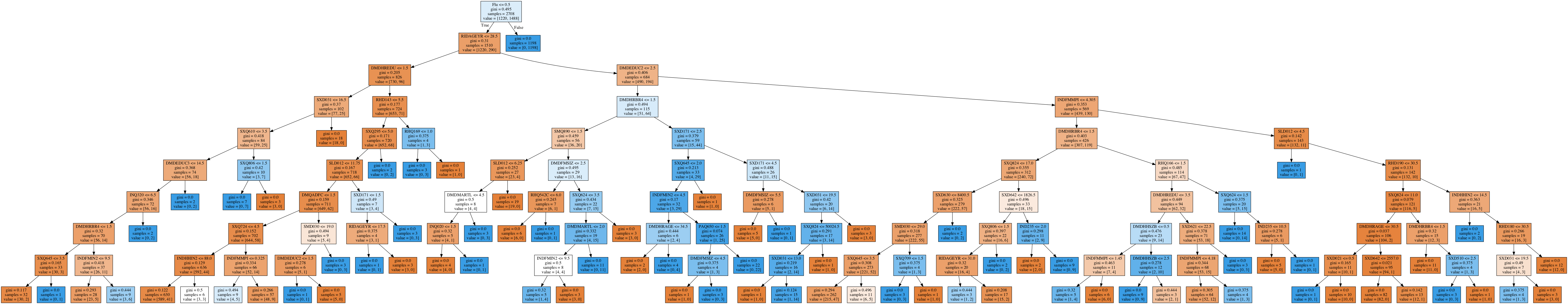

Supplement: Multimedia Appendix 3 [file xmed_v2i2e25560_app3.png]

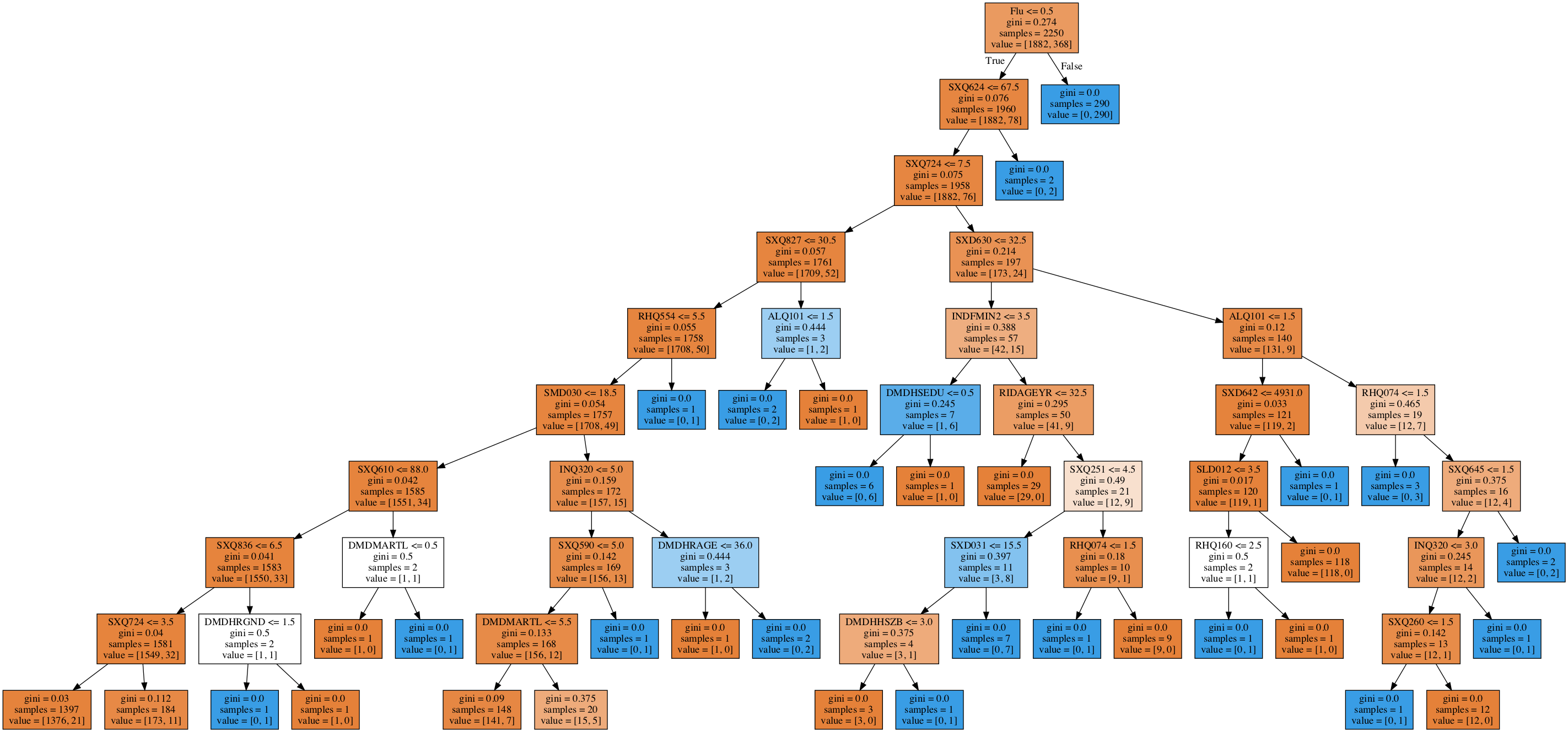

Supplement: Multimedia Appendix 4 [file xmed_v2i2e25560_app4.png]
