# Supplementary material for: Machine Learning for Risk Group Identification and User Data Collection in a Herpes Simplex Virus Patient Registry: Algorithm Development and Validation Study
Source: JMIRx Med. 2021 Jun 11;2(2):e25560. doi: 10.2196/25560 (PMC10414389; doi:10.2196/25560)
Supplement: Multimedia Appendix 5 [file xmed_v2i2e25560_app5.docx]

Multimedia Appendix 4. Set of questions selected by the model

1. The algorithm


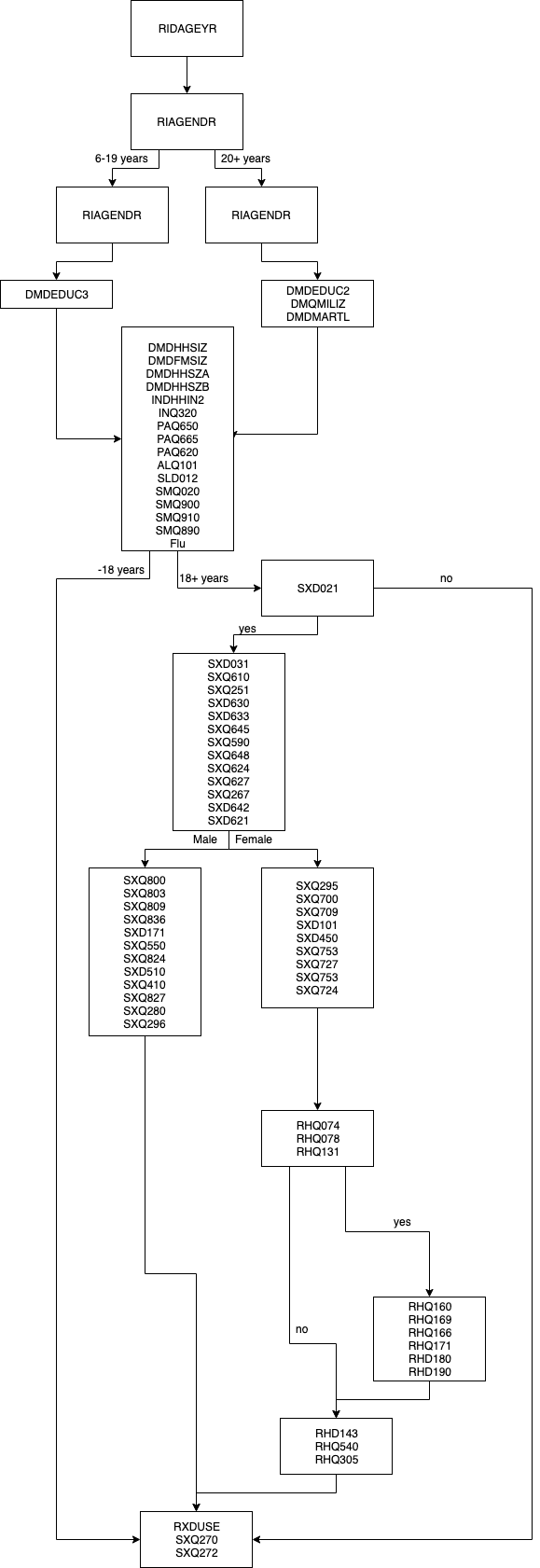


1. List and labels

- 'RIAGENDR': Gender of the participant.,
  - 1 - male, 2-female, 0-missing
- 'RIDAGEYR': Age in years of the participant at the time of screening. Individuals 80 and over are topcoded at 80 years of age.',
  - 0 to 79 - Range of Values, 80 - 80 years of age and over, 0 -missing
- 'DMQMILIZ': Have you ever served on active duty in the Armed Forces, military Reserves, or National Guard?,
  - 1 - yes, 2-no, 0-missing
- 'DMDEDUC3': What is the highest grade or level of school you have completed or the highest degree you have received?,
  - 0-Never attended, 1-...-12 - 1st grade-...-12 grade, 13-High school graduate, 14-GED or equivalent, 15-More than high school, 0 - missing
- 'DMDEDUC2': What is the highest grade or level of school you have completed or the highest degree you have received?,
  - 1-Less than 9th grade, 2 - 9-11th grade (Includes 12th grade with no diploma), 3-High school graduate/GED or equivalent, 4-Some college or AA degree, 5-College graduate or above, 0 - missing
- 'DMDMARTL': Marital status,
  - 1-Married, 2-Widowed, 3-Divorced, 4-Separated, 5-Never married, 6-Living with partner, 0 -missing
- 'DMDHHSIZ': Total number of people in the Household,
  - 1-6 - number from 1 to ... 6, 7 - 7 and more, 0 - missing
- 'DMDFMSIZ': Total number of people in the Family,
  - 1-6 - number from 1 to ... 6, 7 - 7 and more, 0 - missing
- 'DMDHHSZA': Number of children aged 5 years or younger in the household,
  - 0-2 from 0 to 2, 3 - 3 or more, 0 - missing
- 'DMDHHSZB': Number of children aged 6-17 years old in the household,
  - 0-2 from 0 to 2, 3 - 3 or more, 0 - missing
- 'INDHHIN2': Total household income,
  - 1-$ 0 to $ 4,999, 2-$ 5,000 to $ 9,999, 3-$10,000 to $14,999, 4-$15,000 to $19,999, 5-$20,000 to $24,999, 6-$25,000 to $34,999, 7-$35,000 to $44,999, 8-$45,000 to $54,999, 9-$55,000 to $64,999, 10-$65,000 to $74,999, 11-$75,000 to $99,999, 12-$100,000 and Over, 0 -missing
- 'INDFMIN2':'Total family income,
  - 1-$ 0 to $ 4,999, 2-$ 5,000 to $ 9,999, 3-$10,000 to $14,999, 4-$15,000 to $19,999, 5-$20,000 to $24,999, 6-$25,000 to $34,999, 7-$35,000 to $44,999, 8-$45,000 to $54,999, 9-$55,000 to $64,999, 10-$65,000 to $74,999, 11-$75,000 to $99,999, 12-$100,000 and Over, 0 - missing
- 'INQ320': How do you usually get to the store where you do most of your grocery shopping?
  - 1-In my car, 2-In a car that belongs to someone I live with, 3-In a car that belongs to someone who lives elsewhere, 4-Walk,5-Ride bicycle,6-Bus, subway or other public transit,7-Taxi or other paid driver, 8-Someone else delivers groceries, 9-Other, 0 - missing
- 'PAQ650': In a typical week do you do any vigorous-intensity sports, fitness, or recreational activities that cause large increases in breathing or heart rate like running or basketball for at least 10 minutes continuously?,
  - 1 - yes, 2-no, 0-missing
- 'PAQ665': In a typical week do you do any moderate-intensity sports, fitness, or recreational activities that cause a small increase in breathing or heart rate such as brisk walking, bicycling, swimming, or volleyball for at least 10 minutes continuously?,
  - 1 - yes, 2-no, 0-missing
- 'PAQ620': Does your work involve moderate-intensity activity that causes small increases in breathing or heart rate such as brisk walking or carrying light loads for at least 10 minutes continuously?,
  - 1 - yes, 2-no, 0-missing
- 'ALQ101': In any one year, have you had at least 12 drinks of any type of alcoholic beverage? By a drink, I mean a 12 oz. beer, a 5 oz. glass of wine, or a one and a half ounces of liquor.,
  - 1 - yes, 2-no, 0-missing
- 'SXD021': Ever had vaginal, anal, or oral sex?,
  - 1 - yes, 2-no, 0-missing
- 'SXQ800': Have you ever had vaginal sex, also called sexual intercourse, with a woman? This means your penis in a woman's vagina.,
  - 1 - yes, 2-no, 0-missing
- 'SXQ803': Have you ever performed oral sex on a woman? This means putting your mouth on a woman's vagina or genitals.,
  - 1 - yes, 2-no, 0-missing
- 'SXQ809': Have you ever had any kind of sex with a man, including oral or anal?,
  - 1 - yes, 2-no, 0-missing
- 'SXQ700': Have you ever had vaginal sex, also called sexual intercourse, with a man? This means a man's penis in your vagina.,
  - 1 - yes, 2-no, 0-missing
- 'SXQ550': In the past 12 months, with how many men have you had anal or oral sex?,
  - 0 to … - Range of numbers, 0-Missing
- 'SXQ709': 'Have you ever had any kind of sex with a woman? By sex, we mean sexual contact with another woman's vagina or genitals.,
  - 1 - yes, 2-no, 0-missing
- 'SXD031': How old when first had sex?,
  - 13 to 68 - Range of values, 12 - 12 and older, 0 - missing
- 'SXQ295': Describe sexual identity
  - 1-Lesbian or Gay,2-Straight, that is, not lesbian or gay, 3-Bisexual,4-Something else,6-I don't know the answer,7-Refused,9-Don`t know, 0-missing
- 'SXQ836': In your lifetime, with how many men have you had anal sex?,
  - Range of numbers, 100 - 100 or more, 0-missing
- 'SXD171': 'In your lifetime, with how many women have you had any kind of sex?,
  - 0 to 99 - Range of values, 100 - 100 or more, 0 - missing
- 'SXQ824': In your lifetime, with how many women have you had vaginal sex? Vaginal sex means your penis in a woman's vagina.,
  - 0 to 99 - Range of values, 100 - 100 or more, 0 - missing
- 'SXD621': How old were you when you first performed oral sex on a man? Performing oral sex means your mouth on a man's penis or genitals.,
  - 13 to 68 - Range of values, 12 - 12 and younger, 0 - missing
- 'SXD630': How long has it been since the last time you performed oral sex on a new male partner? A new sexual partner is someone that you had never had sex with before.,
  - 0 to 15341 - Range of values, 0-missing
- 'SXQ645': When you performed oral sex in the past 12 months, how often would you use protection, like a condom or dental dam?,
  - 1-Never, 2-Rarely, 3-Usually, 4-Always, 5-Unsure, 0-missing
- 'SXQ267': How old were you when you were first told that you had genital warts?,
  - Range of numbers, 0-missing
- 'SXQ627': In the past 12 months, on how many men have you performed oral sex?,
  - Range of numbers, 0-missing
- 'SXQ590': Of the persons you had any kind of sex with in the past 12 months, how many were five or more years older than you?,
  - Range of numbers, 0-missing
- 'SXD510': In the past 12 months, with how many women have you had any kind of sex?,
  - Range of numbers, 0-missing
- 'SXD633': How old were you when you first performed oral sex on a woman? Performing oral sex means your mouth on a woman's vagina or genitals.,
  - 13 to 50 - Range of values, 12 - 12 and younger, 0 - missing
- 'SXD101': In your lifetime, with how many men have you had any kind of sex?,
  - 0 - 100 Range of numbers, 100 - 100 or more, 0-missing
- 'SXQ648': In the past 12 months, did you have any kind of sex with a person that you never had sex with before?,
  - 1 - yes, 2-no, 0-missing
- 'SXQ624': In your lifetime, on how many women have you performed oral sex? (Male/Female),
  - 0 - 100 Range of numbers, 100 - 100 or more, 0-missing
- 'SXQ610': In the past 12 months, about how many times have you had vaginal or anal sex?,
  - 0-Never, 1-Once, 2-2-11 times, 3-12-51 times, 4-52-103 times, 5-104-364 times, 6-365 times or more, 0-missing
- 'SXQ251': In the past 12 months, about how often have you had vaginal or anal sex without using a condom?,
  - 1-Never, 2-Less than half of the time, 3-About half of the time, 4-Not always, but more than half of the time, 5-Always, 0-missing
- 'SXD450': In the past 12 months, with how many men have you had any kind of sex?,
  - Range of numbers, 0-missing
- 'SXQ727': In the past 12 months, with how many men have you had vaginal sex? Vaginal sex means a man's penis in your vagina.,
  - Range of numbers, 0-missing
- 'SXQ753': Has a doctor or other health care professional ever told you that you had human papillomavirus or HPV?,
  - 1 - yes, 2-no, 0-missing
- 'SXQ410': In your lifetime, with how many men have you had anal or oral sex?,
  - 0-99-Range of numbers, 100-100 or more, 0-missing
- 'SXD642': How long has it been since the last time you performed oral sex on a new female partner? A new sexual partner is someone that you had never had sex with before.,
  - Range of numbers, 0-missing
- 'SXQ270': In the past 12 months, has a doctor or other health care professional told you that you had gonorrhea, sometimes called GC or clap?,
  - 1 - yes, 2-no, 0-missing
- 'SXQ272': In the past 12 months, has a doctor or other health care professional told you that you had chlamydia?,
  - 1 - yes, 2-no, 0-missing
- 'SXQ827': In the past 12 months, with how many women have you had vaginal sex? Vaginal sex means your penis in a woman's vagina.,
  - 0-99-Range of numbers, 100-100 or more, 0-missing
- 'SXQ280': Circumcised or uncircumcised?,
  - 1-Circumcised, 2-Uncircumcised, 0-missing
- 'SXQ636': In your lifetime, on how many women have you performed oral sex?,
  - 0-99-Range of numbers, 100-100 or more, 0-missing
- 'SXQ724': In your lifetime, with how many men have you had vaginal sex? Vaginal sex means a man's penis in your vagina.,
  - Range of numbers, 0-Null
- 'SXQ296': Which of the following best represents how you think of yourself?
  - 1-Gay, 2-Straight that is not gay,3-Bisexual, 4-Something else, 6-I don't know the answer, 0-missing
- 'RXDUSE': In the past 30 days, have you used or taken medication for which a prescription is needed? Do not include prescription vitamins or minerals you may have already told me about.,
  - 1 - yes, 2-no, 0-missing
- 'RHQ074': The next questions are about your pregnancy history. Have you ever attempted to become pregnant over a period of at least a year without becoming pregnant?,
  - 1 - yes, 2-no, 0-missing
- 'RHQ078': Have you ever been treated for an infection in your fallopian tubes, uterus or ovaries, also called a pelvic infection, pelvic inflammatory disease, or PID?,
  - 1 - yes, 2-no, 0-missing
- 'RHQ131': The next questions are about your pregnancy history. Have you ever been pregnant? Please include (current pregnancy,) live births, miscarriages, stillbirths, tubal pregnancies and abortions.,
  - 1 - yes, 2-no, 0-missing
- 'RHQ160': How many times have you been pregnant? (Again/Be sure to count all your pregnancies including (current pregnancy,) live births, miscarriages, stillbirths, tubal pregnancies or abortions.),
  - 1-10 Range of numbers, 11- 11 and more, 0-missing
- 'RHQ169': How many cesarean deliveries have you had? (Cesarean deliveries are also known as C-sections.) (Please count stillbirths as well as live births.)',
  - Range of numbers, 0-missing
- 'RHQ166': How many vaginal deliveries have you had? (Please count stillbirths as well as live births)',
  - 1-10-Range of numbers, 11- 11 and more, 0-missing
- 'RHQ171': How many of your deliveries resulted Did your delivery result in a live birth?,
  - 1-10-Range of numbers, 11- 11 and more, 0-missing
- 'RHD143': Are you pregnant now?,
  - 1 - yes, 2-no, 0-missing
- 'RHD180': How old were you at the time of your first live birth?,
  - 15-43-Range of numbers, 14- 14 and younger, 44-and older, 0-missing
- 'RHD190': How old were you at the time of your last live birth?,
  - 15-43-Range of numbers, 14- 14 and younger, 44-and older, 0-missing
- 'RHQ540': Have you ever used female hormones such as estrogen and progesterone? Please include any forms of female hormones, such as pills, cream, patch, and injectables, but do not include birth control methods or use for infertility.,
  - 1 - yes, 2-no, 0-missing
- 'RHQ305': Had both ovaries removed?,
  - 1 - yes, 2-no, 0-missing
- 'SLD012': How much sleep do you usually get at night on weekdays or workdays?,
  - 2-14.5 Range of numbers, 0-missing
- 'SMQ020': These next questions are about cigarette smoking and other tobacco use. Have you smoked at least 100 cigarettes in your entire life?,
  - 1 - yes, 2-no, 0-missing
- 'SMD030': How old were you when you first started to smoke cigarettes fairly regularly?,
  - 7-58 - Range of numbers, 0-Never smoked cigarettes regularly/missing
- 'SMQ900': The next question is about e-cigarettes. These are battery-powered devices that usually contains liquid nicotine, and don't produce smoke. Have you EVER used an e-cigarette EVEN ONE TIME? This hand card shows examples of some e-cigarettes and other devices used to inhale liquid nicotine; however there are others not included here,
  - 1 - yes, 2-no, 0-missing
- 'SMQ910': Smokeless tobacco products are placed in the mouth and nose and include chewing tobacco, snuff, dip, snus (pronounced as "snoose") and dissolvable tobacco. Have you ever used smokeless tobacco even one time? This hand card shows examples of smokeless products; however there are others not included here.,
  - 1 - yes, 2-no, 0-missing
- 'SMQ890': Have you ever smoked a regular cigar, cigarillo or little filtered cigar even one time? This hand card shows examples of some cigars; however there are others not included here,
  - 1 - yes, 2-no, 0-missing
- 'Flu':Is your general feeling of discomfort or illness followed by one or more symptoms: fever, nausea, headaches, muscle pain, swollen lymph nodes and malaise?
  - 1 - yes, 2-no, 0-missing
