# Supplementary material for: Machine Learning for Risk Group Identification and User Data Collection in a Herpes Simplex Virus Patient Registry: Algorithm Development and Validation Study
Source: JMIRx Med. 2021 Jun 11;2(2):e25560. doi: 10.2196/25560 (PMC10414389; doi:10.2196/25560)

HSV Patient Registry user flows

Legend:

- Suggested plan for beta 1
- Prototyped in Alpha
- Suggested plan for beta 2+

User flows

New user

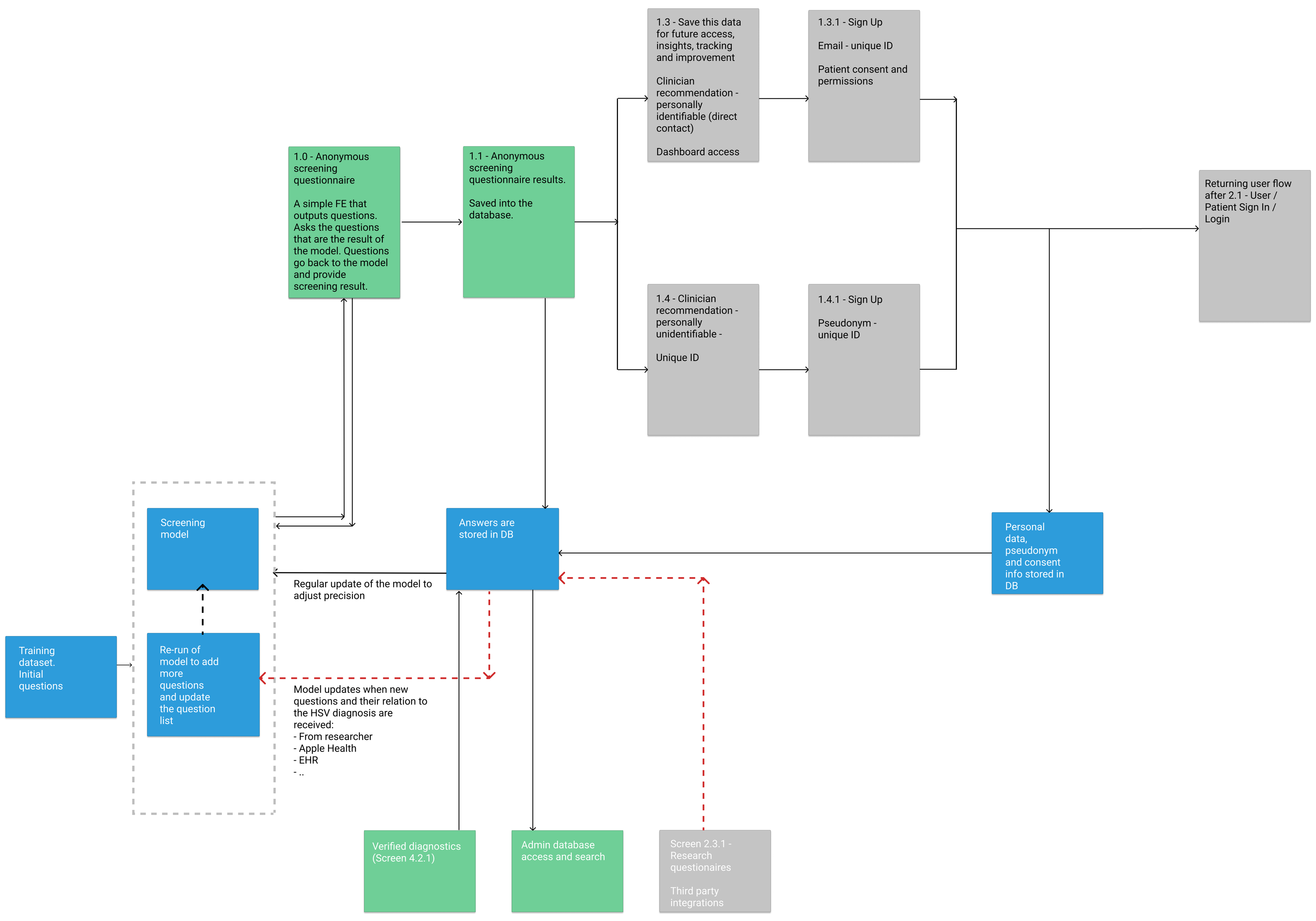

Returning user / Clinician recommendation

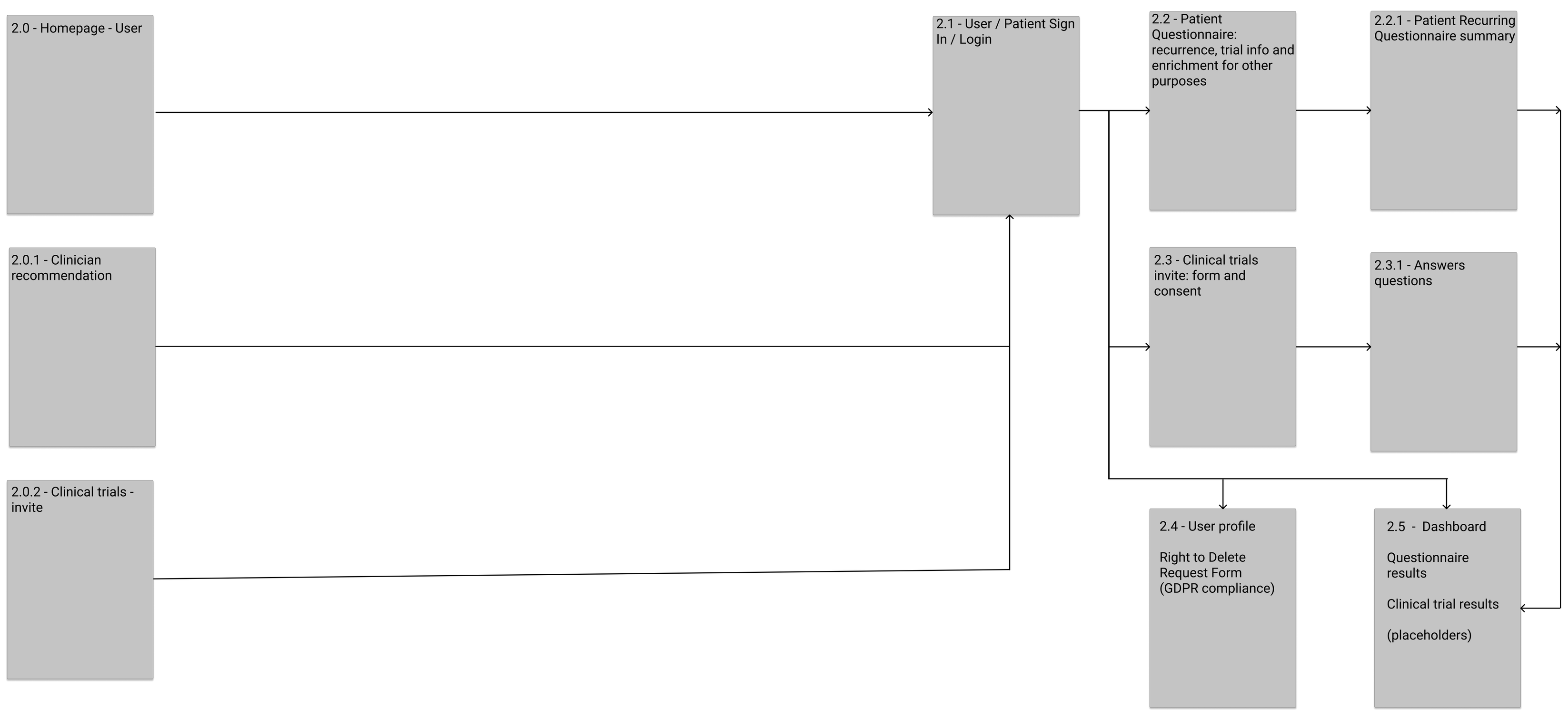

Researcher clinical registration and trial recruitment

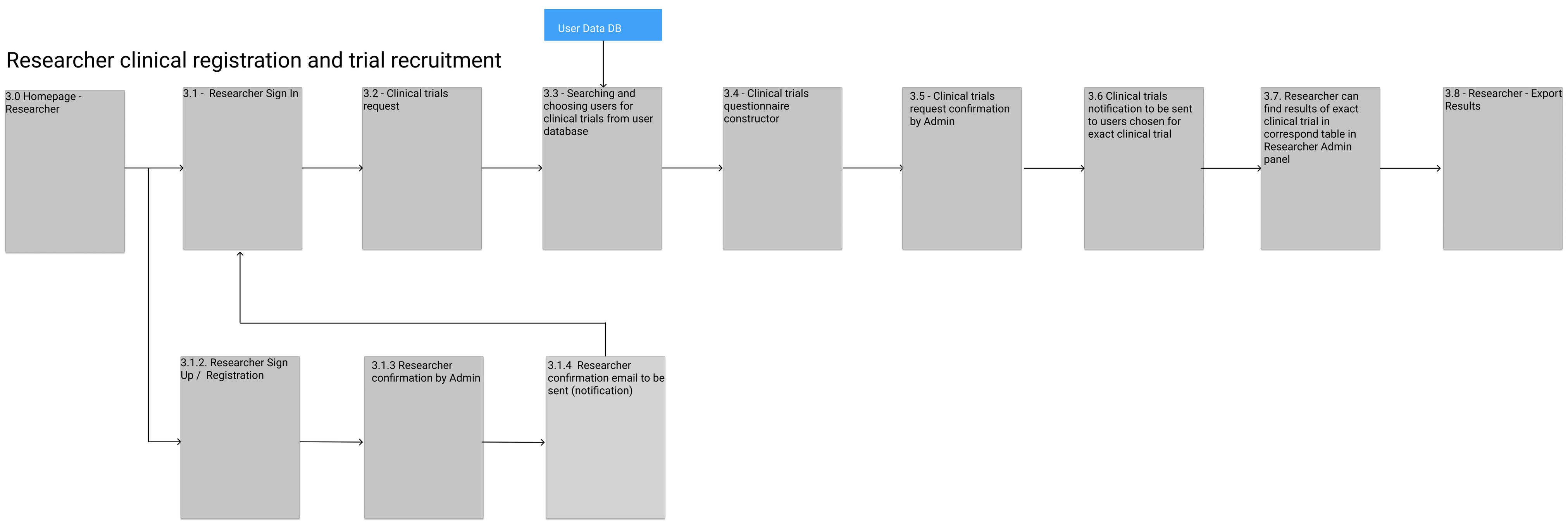

Admin

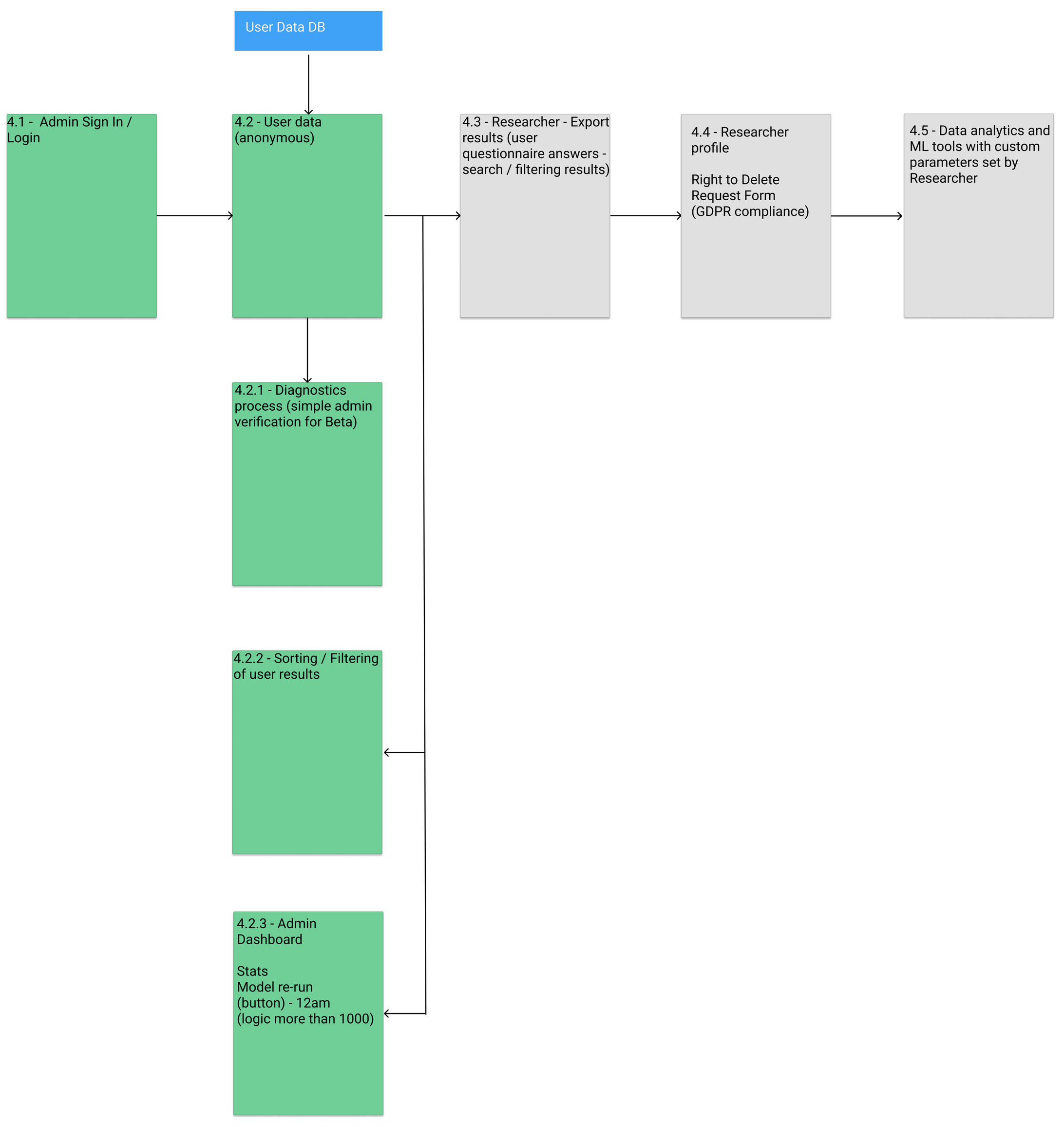

Supplement: Multimedia Appendix 6 [file xmed_v2i2e25560_app6.pdf]
